# On the Verge of the 3000th Publication: Reflections on Deceptions, Successes and Perspectives

**DOI:** 10.3390/molecules31162896

**Published:** 2026-08-20

**Authors:** Erik De Clercq

**Affiliations:** Department of Microbiology, Immunology and Transplantation, Rega Institute for Medical Research, KU Leuven, B-3000 Leuven, Belgium; erik.declercq@kuleuven.be

**Keywords:** DHPA, BVDU, VACV, d4T, emivirine, rilpivirine, HPMPC, PMEA, tenofovir, AMD-3100

## Abstract

A lifelong career devoted to the development of specific antiviral agents eventually yielded, besides (almost) 3000 publications, about ten antiviral compounds that were approved and marketed as antiviral drugs, i.e., DHPA (Duviragel^®^), BVDU (Zostex^®^, Mevir^®^, Brivir^®^…), VACV (Zelitrex^®^, Valtrex^®^), d4T (Stavudine^®^), emivirine (Coactinon^®^), rilpivirine (Edurant^®^), HPMPC (Vistide^®^), PMEA (Hepsera^®^), tenofovir (Viread^®^) and AMD-3100 (Mozobil^®^), with the latter as a hematopoietic stem cell mobilizer. The targeted viruses for these compounds were herpes simplex virus (HSV), varicella-zoster virus (VZV), cytomegalovirus (CMV), human immunodeficiency virus (HIV), and hepatitis B virus (HBV). For other viruses, such as filo-, rhabdo-, arena-, myxo-, polyoma- and papillomaviruses, strategies have been elaborated that should facilitate future developments of antiviral drugs.

## 1. Foreword

As the highlights of my scientific career, I look back at the interferon research I started at the Rega Institute with Prof. Piet De Somer in Leuven (Belgium) and then continued with Prof. Thomas C. Merigan at Stanford University. Interferon was originally contemplated as a broad-spectrum antiviral agent, although in the mid-70s, Dr. Mathilde Krim emphasized its antitumor potential.

The discovery of the reverse transcriptase (RT) by Howard Temin and David Baltimore in 1970 (later recognized by the Nobel Prize to Baltimore, Dulbecco and Temin in 1975) generated great expectations of the RT in the origin of cancer (Sol Spiegelman). It made me dream of suramin as a potential anticancer agent when I had found it to be a potent RT inhibitor. I told Dr. Robert Gallo about this finding (in 1975) when he visited me (in 1978). Suramin did not fulfill its promise as an antitumor agent, but it became the first anti-HIV drug to be attempted in the fight against AIDS.

Then followed a variety of nucleoside RT inhibitors (NRTIs) described by Dr. Hiroaki Mitsuya and Dr. Sam Broder, and D4T (Stavudine^®^) described by ourselves (Dr. Masanori Baba et al.).

That non-nucleosides would ever behave as RT inhibitors (NNRTIs) was unexpected. This became evident with the TIBOs (Dr. Rudi Pauwels, Dr. Paul Janssen), which later led to the discovery of rilpivirine, and HEPTs (Dr. Masanori Baba), which later yielded emivirine (Coactinon^®^) before its further (clinical) development was ended.

Serendipitously, AMD-3100 was discovered as an anti-HIV compound. It turned out to be a CXCR4 antagonist (Prof. Dominique Schols) and found its application as a hematopoietic stem cell mobilize (plerixafor, Mozobil^®^) in the treatment of non-Hodgkin lymphoma and multiple myeloma.

## 2. Introduction

During the first year of my medical studies, I was once invited for dinner at the home of Prof. Xavier Aubert (Professor of Physiology at the French section of KU Leuven), who was married to the oldest daughter of Prof. Abert Dalcq (Professor of Anatomy at the Université Libre de Bruxelles, who would become Secrétaire Perpétuel of the French section of the Belgian Royal Academy of Medicine). At the dinner party, Prof. Aubert advised me that I should do some research work in a laboratory of my choice. When I told him that I preferred biochemistry, he pointed out that the Professor of Biochemistry at the Flemish section (KU Leuven), Prof. Paul Putzeys, did not accept medical students to work in his laboratory but that his counterpart in the French section, a certain Christian de Duve, did so. When I inquired what kind of work Christian de Duve was doing, he told me this work was related to cell compartments, which I did not find very tempting, so I missed the opportunity to collaborate with a future Nobel laureate. Instead, my interest directed me to chimie hormonologique, the specialty of Dr. Raymond Devis. What I dreamed of was the biosynthesis of steroid hormones, but instead I had to settle for the analysis of catecholamines by thin layer chromatography and spectroscopy, and for several years (second, third and fourth year of my medical training), I spent all my afternoons in the laboratory of chimie hormonologique. Although Dr. Devis ostensibly appreciated my achievements, they did not lead to any reports or publications, so that I cannot prove by any documents that I ever got any training in chimie hormonologique (the chemistry of hormones).

## 3. Rubella Virus Vaccine

I had been asked by Prof. Piet De Somer (PDS) at the microbiology exam in the fourth year of medical school to join his research group, but because I expected this research to be focused on bacteriology, I did not show great enthusiasm. When I told Olaf Leuridan about my unwillingness to join PDS’ research team, the president of the medical students’ union, he told me it was a stupid decision not to accept an invitation from the most important person in the academic world in Leuven, and when Prof. De Somer repeated his invitation at the virology exam in the fifth year, I duly responded with an unconditional “yes”. The sixth year of medical school was crammed with internships (internal medicine, surgery, obstetrics, neurology, and radiotherapy), leaving no time for research. In the seventh year, I accomplished some master’s work on the development of a rubella vaccine (a thesis for the PhD of Prof. Jan Desmyter), which excited PDS when I told him that with the Cendehill strain of the rubella virus I had obtained an antibody titer (by seroneutralization) of ≥10,000. He uttered that this was the beginning of the vaccine, and, although I did not understand his reaction, he was correct; the Cendehill strain later became a rubella virus vaccine.

## 4. Polyanionic Inducers of Interferon

After graduating as a medical doctor (MD) in July 1966, I started my PhD research work in August 1966 and promptly found that polyacrylic acid (PAA) was able to induce interferon. This observation sparked the interest of PDS again, and he talked about these findings at a meeting in Fort Lauderdale (Florida, USA). The results were finally published in two consecutive papers in the *Journal of Virology* [1,2] and a third one in *Applied Microbiology* [3]. At the Fort Lauderdale meeting, Prof. De Somer also talked to Prof. Thomas C. Merigan (TCM), from whom he learned that TCM had discovered in collaboration with Dr. W. Regelson pyran copolymer as an inducer of interferon [4,5].

PDS returned from Fort Lauderdale with the advice that I should spend some time in the US to get acquainted with the American lifestyle. As possible choices, he recommended (*i*) TCM (Stanford, Palo Alto), (*ii*) Bob Wagner (Johns Hopkins, Baltimore) or (*iii*) Phil Marcus (Bronx, New York City). The fact that TCM was involved in the study of polyanions similar to PAA, and was located in Palo Alto, influenced my decision to opt for Stanford, so that on 3 September 1968, after we had been married on 31 August, the newlyweds (Lili Declercq and I) undertook a long flight from Brussels, via London, Los Angeles, and San Francisco, to Palo Alto, the last segment via helicopter, to finally settle for our honeymoon in the Rickeys Hyatt resort hotel (which no longer exists) at El Camino Real in Palo Alto. The next morning, on 4 September 1968, I was introduced by Prof. Merigan to the medical school of Stanford University.

## 5. Life at Stanford University Medical School

After I had become accustomed to the Stanford environment, I identified some aspects of the mechanism of action of synthetic polynucleotides, which made Prof. Merigan so excited that he arranged a visit to Prof. Paul Berg of the biochemistry department (and member of the US National Academy of Sciences). The intention was to ask Dr. Berg to sponsor an envisaged paper for publication in the *Proceedings of the National Academy of Sciences* (PNAS). Paul Berg found the results quite interesting, but still premature for publication in PNAS. On the way back from Dr. Berg’s office to TCM’s laboratory, when TCM had realized that the paper was not going to be published in PNAS, he almost instantly decided to submit the paper for publication to *Nature*. I thought that TCM’s decision was temerarious, but I later shared his joy when *Nature* accepted the paper (without revision) for publication [6]. Shortly thereafter it was followed by another article published in *Science* [7] on the interferon-inducing capacity of thiophosphate-substituted polynucleotides [i.e., poly r($\bar{s}$A-$\bar{s}$U)]. The articles in *Nature* [6] and *Science* [7] heralded a most productive (and delightful) stay at the Stanford University Medical School, reflected by many more publications in distinguished journals, i.e., *Journal of Clinical Investigation* (JCI) [8] and *Journal of Molecular Biology* (JMB) [9], than would be required for any PhD thesis. However, I had not formally been enrolled for obtaining such a degree, and, accordingly, I did not apply for a PhD degree at Stanford University.

## 6. Poly(I)∙Poly(C) and the Cloning of Interferon

Poly(I)∙poly(C) was first published in 1967 [10] as representative for a whole series of double-stranded RNAs as inducers of interferon. It was recognized as the most potent interferon inducer among a broad array of substances [11]. It is a pity that poly(I)∙poly(C) despite its promise [8] was never developed as a clinical therapeutic. That the interferon inducing capacity of poly(I)∙poly(C) could not be dissociated from its toxic side effects may have contributed to this failure. Yet, poly(I)∙poly(C) proved critically useful in the induction of interferon mRNA, which allowed the cloning and expression of human fibroblast interferon [12,13]. As a side product, it also allowed the identification of interferon β2 [14] that later would be better known as interleukin-6.

## 7. Suramin as Inhibitor of the RT (Reverse Transcriptase; RNA-Dependent DNA Polymerase)

Shortly before my return from Stanford University, the RT was discovered by Temin and Mizutani [15] and Baltimore [16]. Upon my return to the Rega Institute, I was extremely delighted to confirm this finding, which allowed me to search for potential inhibitors of this enzyme. I quickly identified suramin (Moranyl) as a potent RT inhibitor (in 1975).

Although the RT was originally linked to the replication of murine retroviruses, Sol Spiegelman and colleagues detected the enzyme in all sorts of cancer, which led the scientific community to believe that the RT had some role in the pathogenesis of cancer. Accordingly, I treated mice that had been injected with murine leukemic cells with suramin, in the hope that suramin would inhibit the leukemic tumor growth and prolong the life span of the mice. Unfortunately, this outcome did not happen. I told Bob Gallo of this failure when he visited me sometime in 1978, and Gallo, who had in the meantime become the Editor (-in-Chief) of *Cancer Letters*, invited me to submit the RT (without the leukemia) data on suramin to his journal. The paper was accepted by *Cancer Letters* (without the need for revision) [17].

The hype about the role of RT in cancer waned and I forgot about my original experience with suramin until in 1984 I received (at home) a sudden phone call from Dr. Sam Broder to compliment me with the discovery of suramin as an RT inhibitor. This discovery had apparently inspired Broder, Gallo and their colleagues to try suramin (with success) as an inhibitor of HIV (then called HTLV-III) replication [18]. Furthermore, Dr. Broder invited me to Washington DC where on 2 June 1985 a meeting would be held at NIH, to discuss the future of the potential treatment of HIV (HTLV-III) infections.

## 8. Azidothymidine (AZT, 3′-Azido,2′,3′-dideoxythymidine, Zidovudine, Retrovir^®^) (Figure 1) as HIV (HTLV-III) Inhibitor

The NIH meeting of 2 June 1985 was formally focused on the in vitro activity of suramin against HTLV-III; the in vivo efficacy was published in *The Lancet* later that same year [19]. In his talk, Sam Broder referred to a new compound that the NIH (NCI) had obtained from Burroughs Wellcome (BW) and that proved more effective. The compound was only mentioned by its code name: BW A509U. The whole audience was looking at Dave Barry (from BW), but he remained astonishingly enigmatic, stating that he knew the identity of the compound, but was not going to reveal it, except that it was a nucleoside analog but not “acyclovir”. We had to wait until the October issue of PNAS had appeared, in which the compound was announced as azidothymidine [20]. In retrospect, azidothymidine was not a new compound. Its synthesis had already been described by Jerome Horwitz and his colleagues [21], and I had already evaluated the antiviral activity of the compound and several other nucleoside analogs, but unfortunately I had not done so for its inhibitory activity against retroviruses (for which I had elaborated the required in vitro [17] and in vivo assays [22]). In this case, I should have detected the potential activity of AZT against retroviruses.

**Figure 1:**
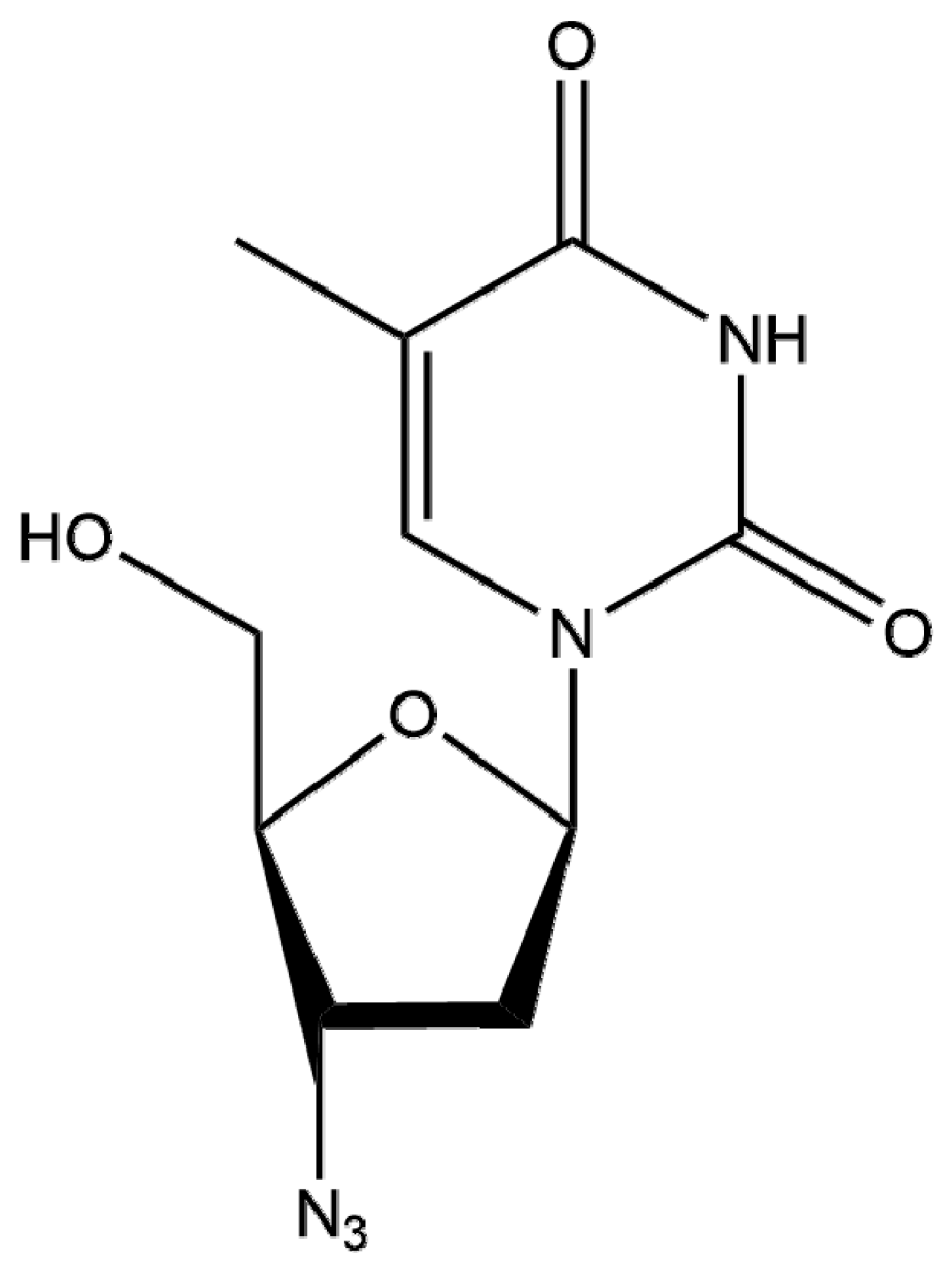
AZT, Retrovir^®^.

## 9. Other 2′,3′-Dideoxynucleoside (ddN) Nucleoside Analogs as HIV Inhibitors (Figure 2, Figure 3, Figure 4, Figure 5, Figure 6 and Figure 7)

**Figure 2:**
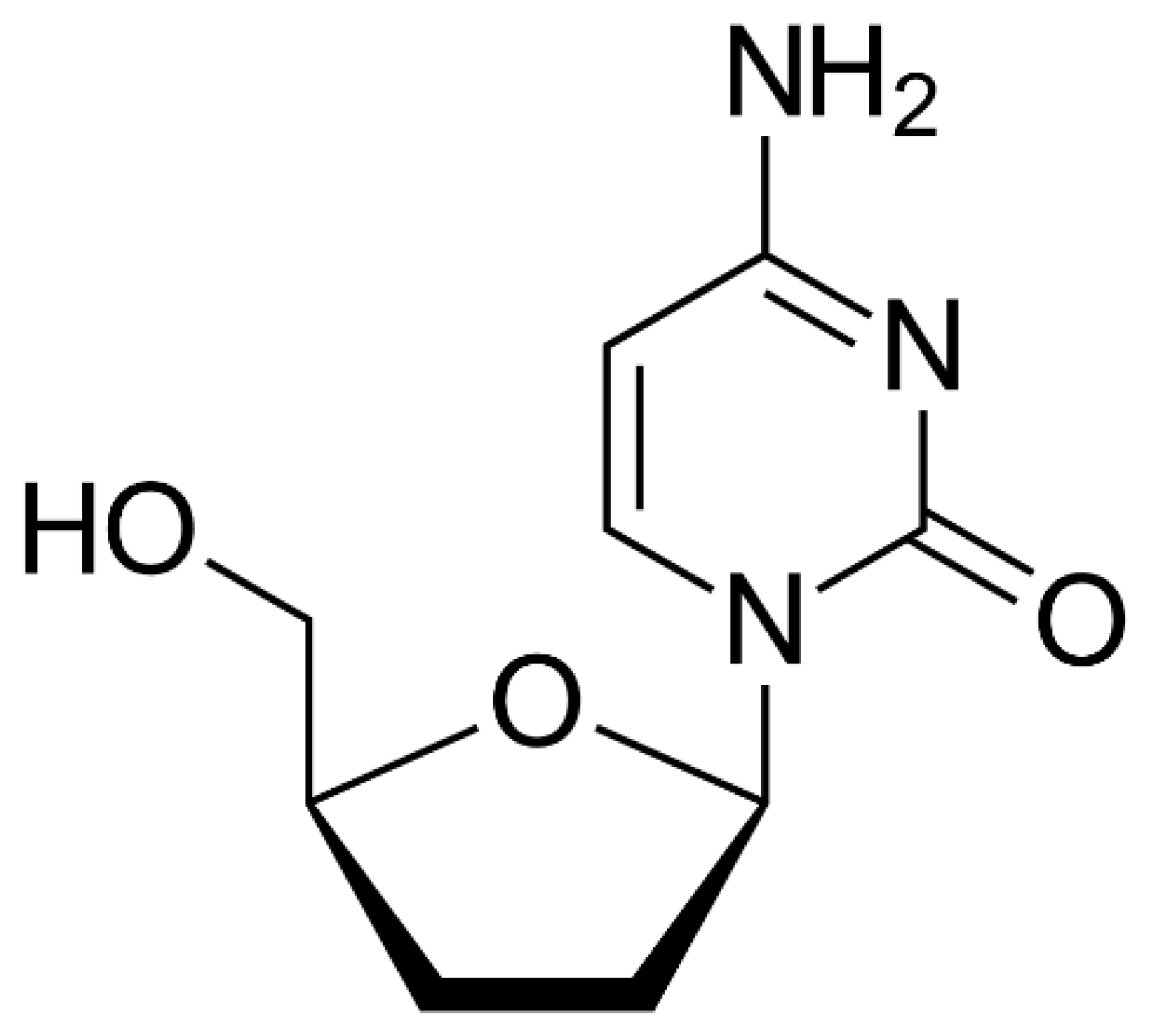
ddC, zalcitabine.

**Figure 3:**
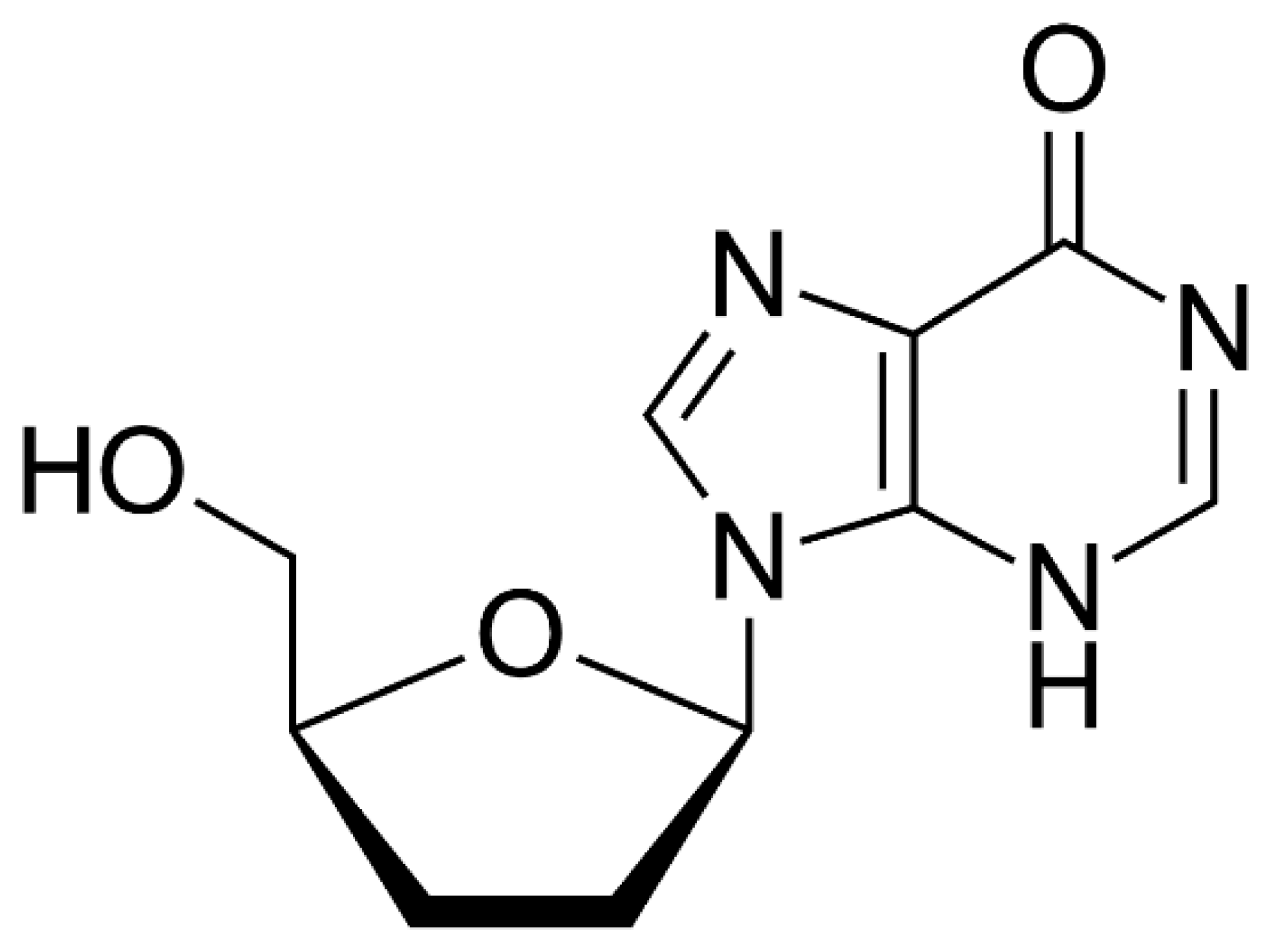
ddI, didanosine.

**Figure 4:**
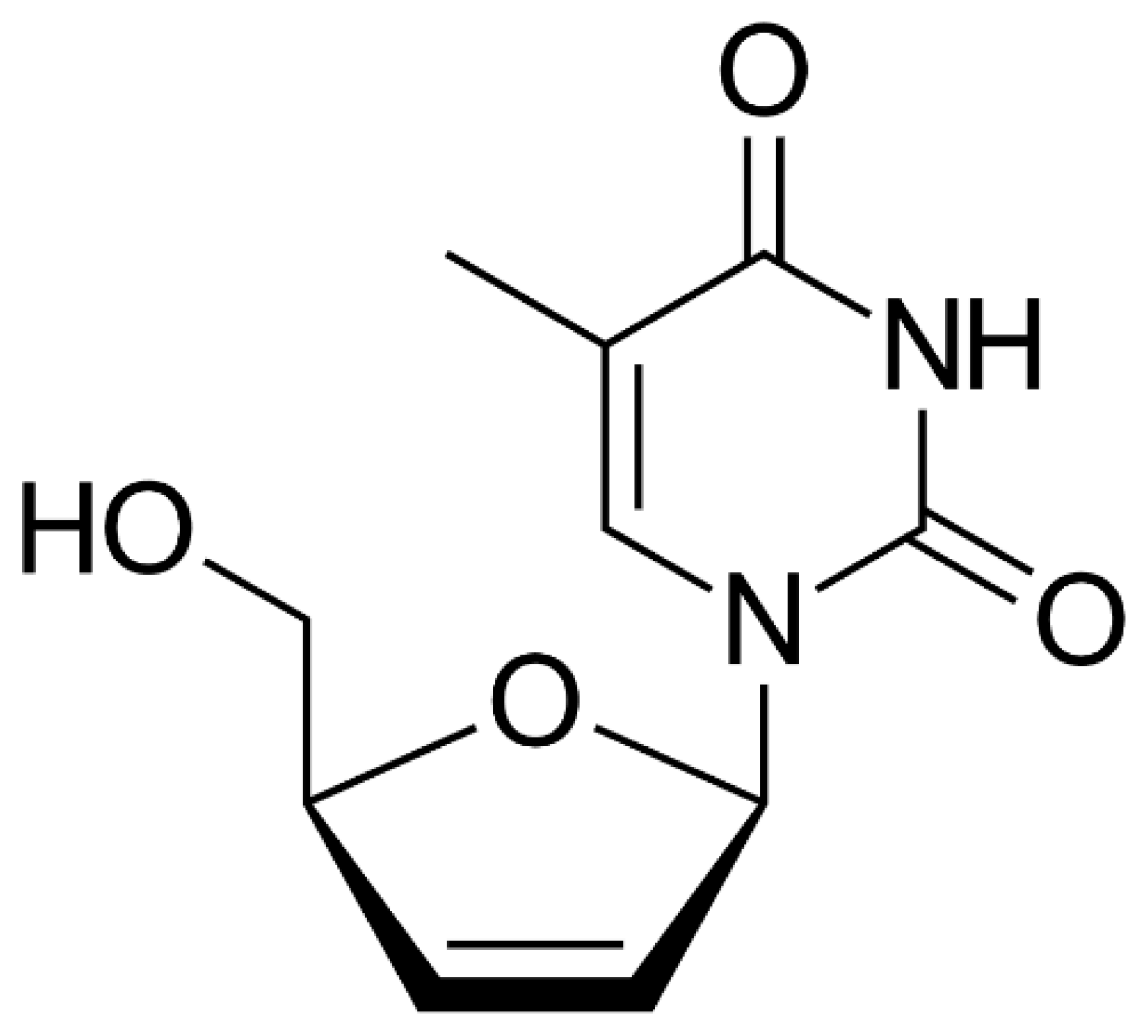
d4T, Stavudine^®^.

Following AZT, several other 2′,3′-dideoxynucleoside analogs such as 2′,3′-dideoxycytidine (ddC) (Figure 2) and 2′,3′-dideoxyinosine (ddI) (Figure 3) were reported to inhibit the replication of HTLV-III [18]. The 2′,3′-dideoxy-,2′,3′-didehydrothymidine (d4T) (Figure 4), originally referred to as 2′,3′-dideoxythymidinene, was synthesized, as were several other 2′,3′-dideoxy nucleoside analogues, by Dr. Piet Herdewijn at the Rega Institute. They were sent in early 1986 for further evaluation for activity against HTLV-III to Jan Balzarini, who was then working at NCI in Sam Broder’s laboratory. Employing the ATH8 cell culture, mediocre activity against HTLV-III was found with d4T, so that no attempts were made to patent and/or publish these findings. The situation changed with the arrival of Dr. Masanori Baba in our institute in August 1986. We were now working with the MT-4 cells, which we had obtained from Dr. Naoki Yamamoto, with the help of Dr. Luc Montagnier, and Masanori Baba found HTLV-III (HIV) to be exquisitely sensitive to d4T in the MT-4 cells. This resulted in a paper that we submitted for publication to Dr. Alberto Sols in November 1986.

The paper was received on 25 November 1986 and published in *Biochemical and Biophysical Research Communications* (BBRC) [23] on 15 January 1987. At the same time that we submitted the paper for publication to Dr. Sols (Editor of *Biochemical and Biophysical Research Communications*), we sent the paper for filing patent protection to our patent attorney Mr. C.W. Bruin (Arnold & Siedsma) in Den Hague (the Netherlands), but Mr. Bruin forgot (or ignored) to file the paper for patent protection. Later we learned that Prof. W.H. Prusoff had filed patent protection for d4T sometime in December 1986. Although our patent rights on d4T (Stavudine^®^) were denied, we could find some solace in the fact that we were 6 months earlier than our competitors in publishing d4T as an anti-HIV agent [23,24,25].

**Figure 5:**
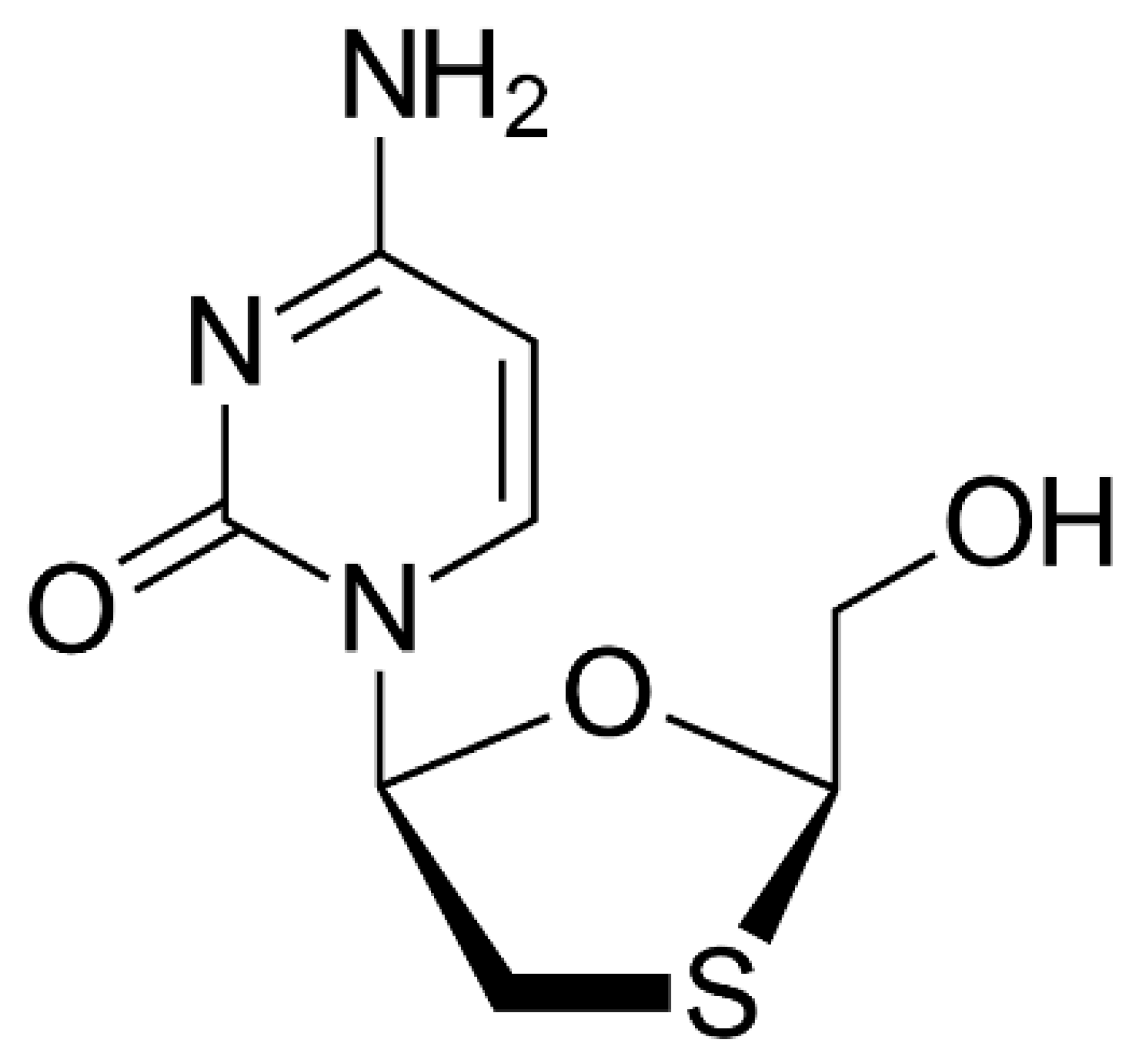
3TC, lamivudine, Epivir^®^.

**Figure 6:**
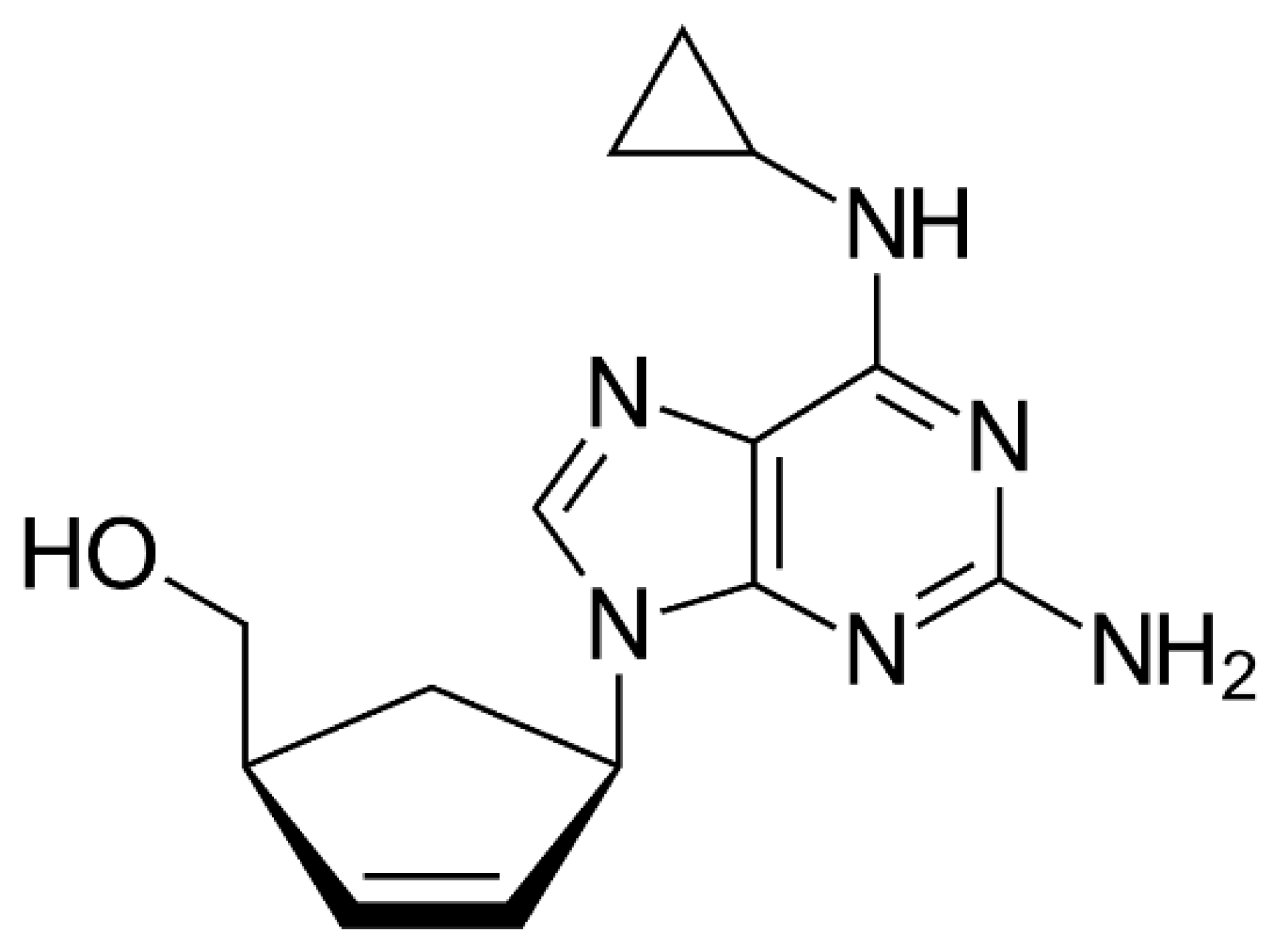
ABC, abacavir, Ziagen^®^.

**Figure 7:**
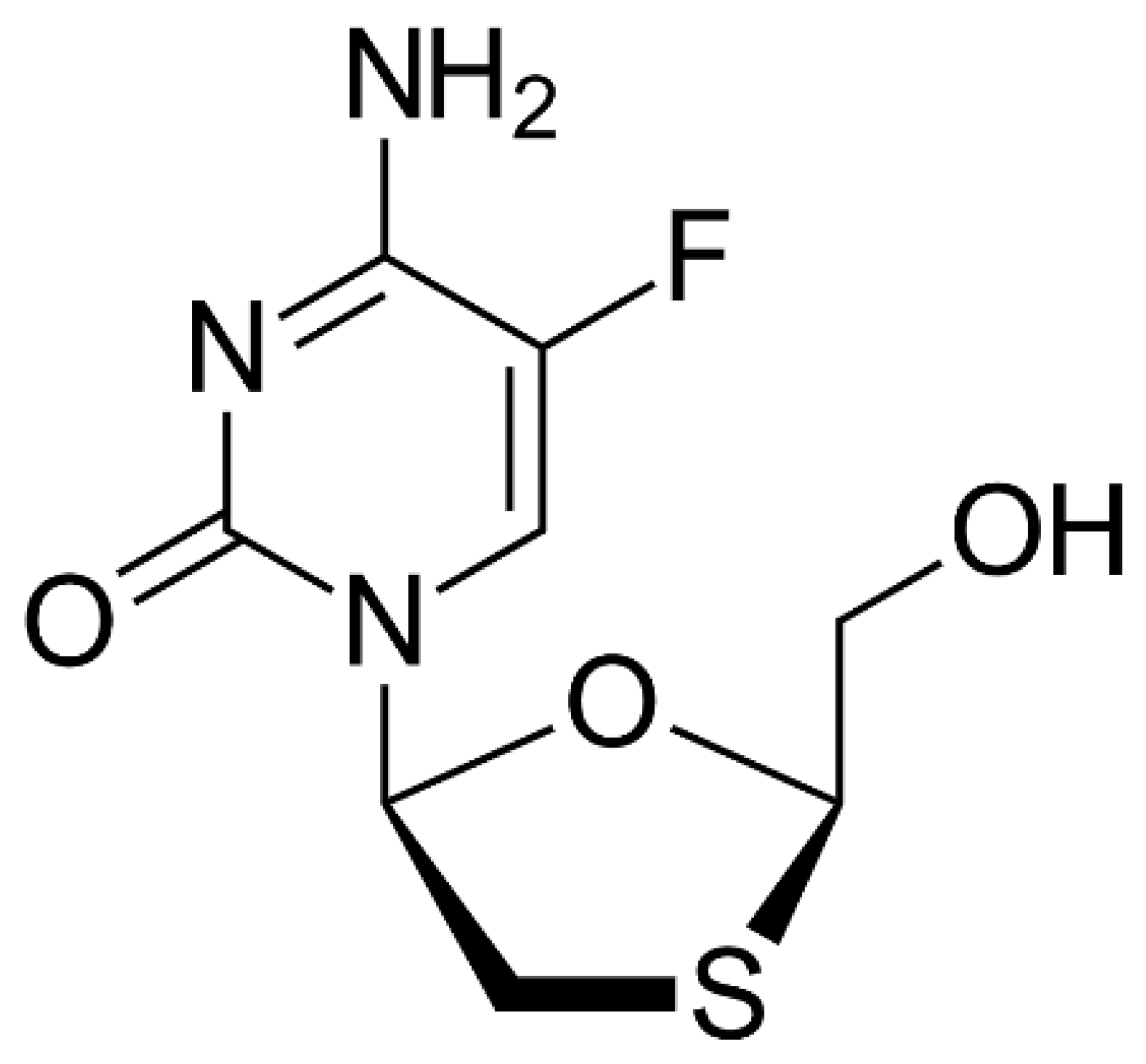
(−)FTC, emtricitabine, Emtriva^®^.

Following ddC, ddI and d4T, several other ddN analogs were described as HIV inhibitors, i.e., 3TC (Figure 5) [26], ABC (Figure 6) [27] and (−)FTC (Figure 7) [28].

## 10. Aminoacyl Esters of Acyclovir (Figure 8)

The discovery of the activity of acyclovir against herpes simplex virus (HSV) has been attributed to Peter Collins in Steve Bauer’s laboratory in 1974 (Wellcome, Beckenham, UK) [29], and Dr. Gertrude B. Elion described the role of the HSV-encoded thymidine kinase (TK) in the phosphorylation of acyclovir in December 1977 [30], a few months before the anti-HSV activity of acyclovir was documented [31]. The marked activity of acyclovir against HSV [both type 1 (HSV-1) and type 2 (HSV-2)] prompted Leon Colla in the laboratory of Prof. Hubert Vanderhaeghe (Rega Institute) to synthesize the amino acid (i.e., glycine and alanine) esters of acyclovir, essentially with two aims: (*i*) to make acyclovir available as eye drops (instead of eye ointment) for the topical treatment of HSV keratitis and (*ii*) to formulate acyclovir for intramuscular administration (a goal that was never realized). The first goal was achieved with the glycine ester of acyclovir, which proved indeed efficacious in the topical treatment, as eye drops, of HSV keratitis in rabbits [32]. The patent rights on the antiviral activity of the aminoacyl esters of acyclovir were transferred from the Rega Institute to Burroughs Wellcome, later incorporated into GlaxoSmithKline (GSK), and one of the aminoacyl esters, i.e., valacyclovir (Valtrex^®^, Zelitrex^®^), then replaced acyclovir for the systemic treatment of HSV infections as its oral bioavailability superseded that of the parent compound.

**Figure 8:**
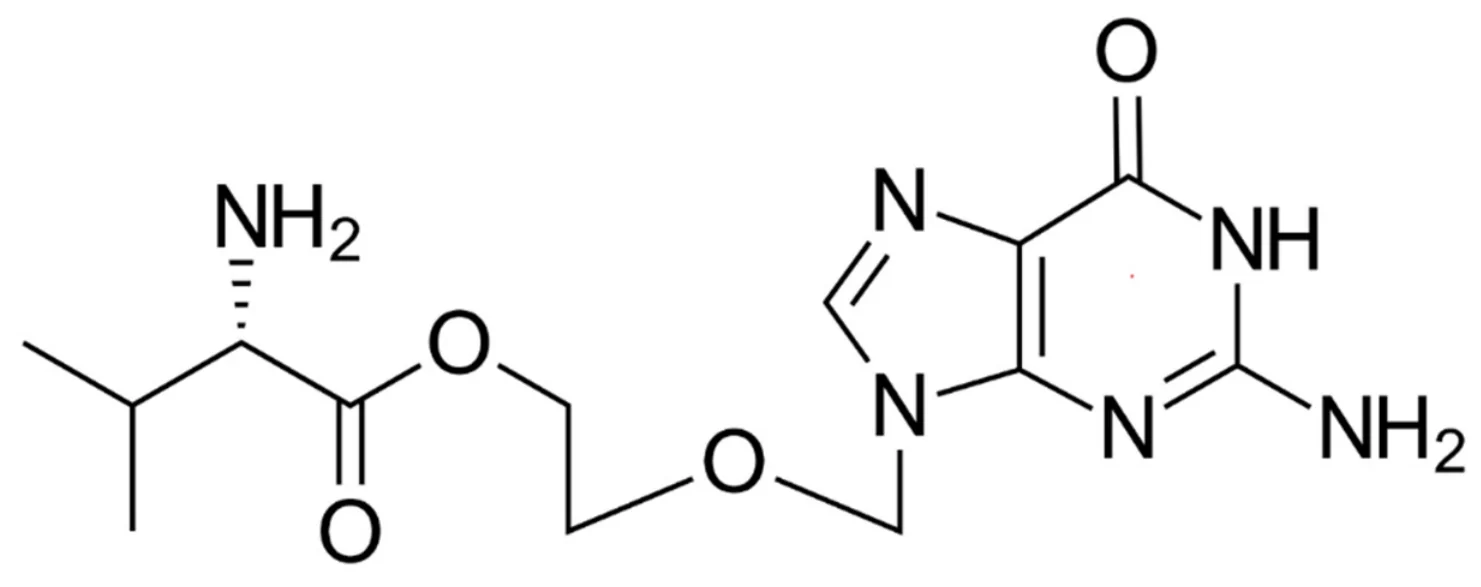
Valacyclovir (VACV), Zelitrex^®^, Valtrex^®^.

## 11. (E)-5-(2-Bromovinyl)-2′-deoxyridine, Brivudine, BVDU (Figure 9)

In May 1976 (at the Symposium on Synthetic Nucleosides, Nucleotides and Polynucleotides, Max-Planck-Institut für Biophysikalische Chemie, Göttingen, Germany, 3–5 May 1976), I met Dr. R.T Walker, Dr. A. Holý and several other chemists. It was the start of a collaborative study of the antiviral activity of nucleoside analogs synthesized in Dr. Walker’s laboratory. From this study, BVDU emerged as a selective and potent inhibitor of HSV-1 replication [33]. One year later, we published on five patients with varicella-zoster virus (VZV) infection who responded favorably to treatment with BVDU [34]. Surprisingly, BVDU was much less active against HSV-2 than HSV-1 [35], apparently because the HSV-1-encoded TK was able to further phosphorylate the monophosphate to the diphosphate (thus facilitating the formation of the triphosphate), whereas the HSV-2-encoded TK was devoid of this propensity [36]. That BVDU discriminated between HSV-1 and HSV-2 was the main reason for the company Searle to stop further clinical development of BVDU in 1984 and to return the patent rights to its legal owners (Rega Institute and Birmingham University, UK). Then followed a long series of attempts to get other companies interested in BVDU, but they invariably failed. The only positive signal came from a company, i.e., Berlin Chemie in the former DDR, that had commercialized BVDU (Brivudine) as Helpin^®^, for the treatment of VZV infections, i.e., herpes zoster, in immunocompromised patients. Investigators of Berlin-Buch, led by Dr. Peter Langen, had initially mistaken BVDU for (*E*)-5-(1-bromovinyl)-2′-deoxyuridine, and this made their patent claims invalid when in 1989 East Germany (DDR) and West Germany were united again. Meanwhile, Berlin Chemie had been taken over by the Italian company Menarini, and Dr. Antonio Giachetti from Menarini then secured the approval of BVDU (Brivudine) for the treatment of herpes zoster in immunocompetent patients. This led to the new start of BVDU under different trade names (Zostex^®^, Brivirac^®^, Zerpex^®^, Mevir^®^, Brivir^®^…) in different countries across the globe.

**Figure 9:**
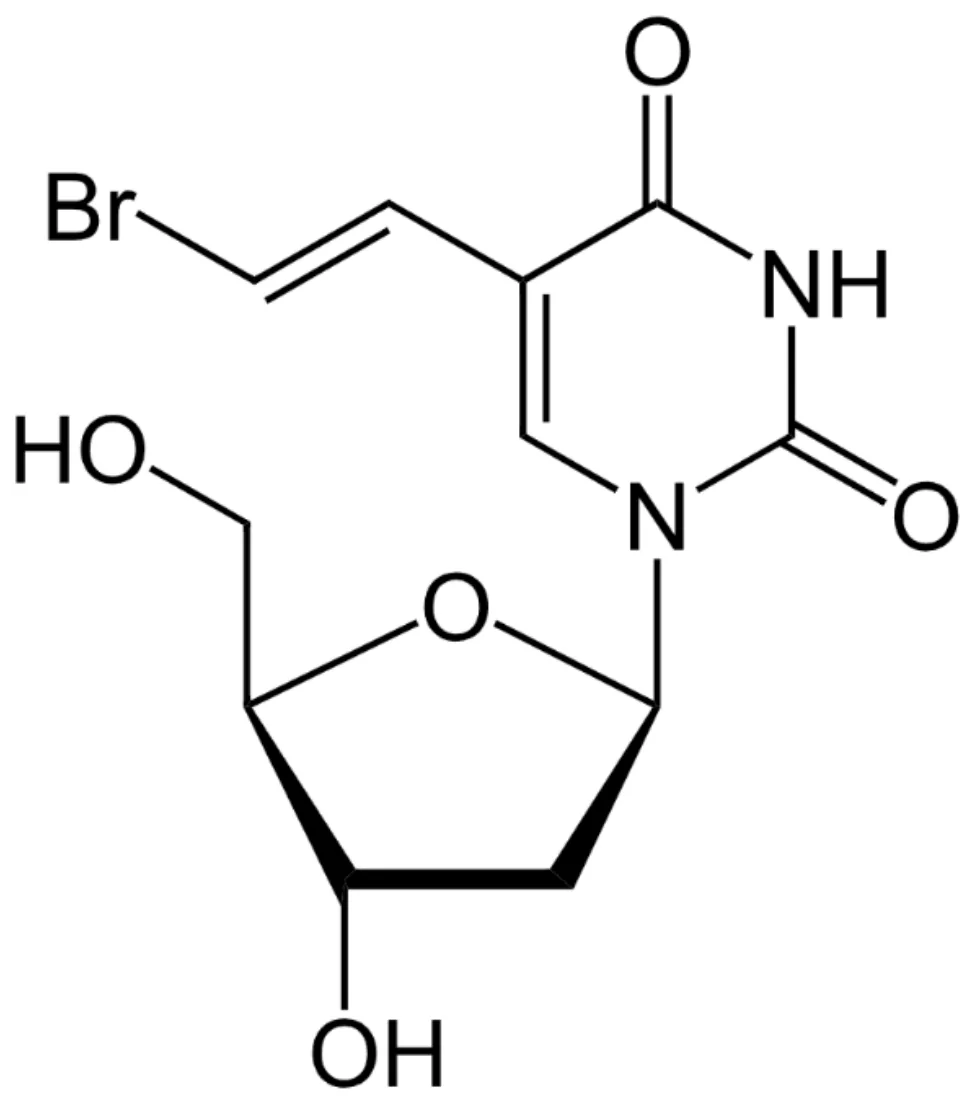
Brivudine (BVDU), Zostex^®^, Brivirac^®^, Zerpex^®^, Mevir^®^, Brivir^®^….

## 12. *S*-2′,3′-Dihydroxypropyl Adenine (DHPA) (Figure 10)

At the Göttingen meeting, I also met with Dr. A. Holý, initiating the start of a new collaboration originally involving three compounds. One of the three compounds turned out to be antivirally active against some viruses, i.e., vaccinia virus (VV) and vesicular stomatitis virus (VSV) [37]. Although no data were reported for this compound against HSV, the compound was marketed as Duviragel^®^ cream for the local treatment of herpes labialis by a Czechoslovak company, Lachema. With the dissolution of Czechoslovakia into the Czech Republic and the Slovak Republic, Lachema disappeared, and so did Duviragel for the treatment of herpes labialis (cold sores).

**Figure 10:**
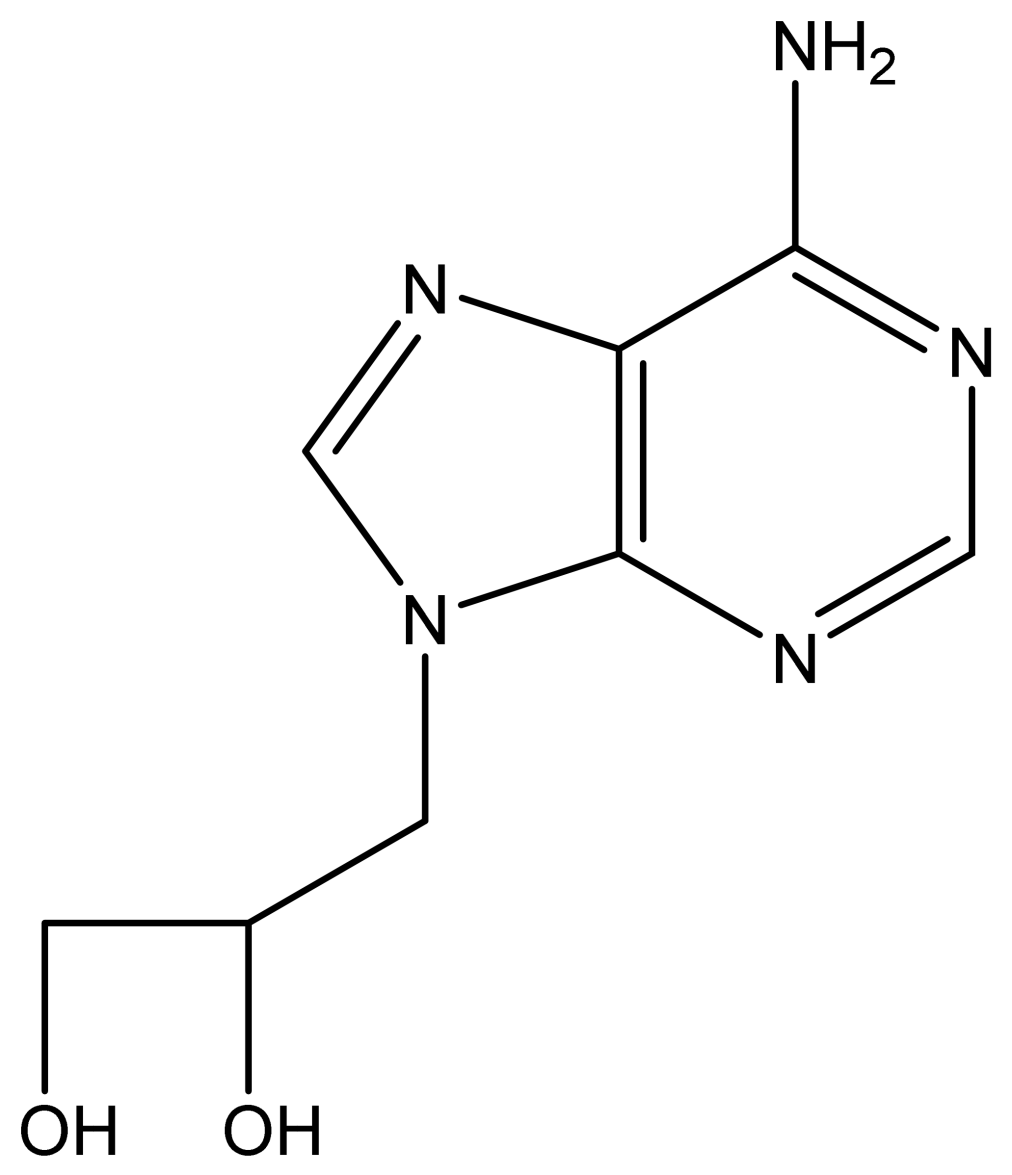
DHPA, Duviragel^®^.

## 13. The Acyclic Nucleoside Analogs (S)-9-(3-Hydroxy-2-phosphonylmethoxypropyl)adenine [(S)-HPMPA] (Figure 11), (S)-1-[3-Hydroxy-2-phosphonylmethoxypropyl]cytosine [(S)-HPMPC] (Figure 12) and 9-[2-(Phosphonomethoxy)ethyl]adenine (PMEA) (Figure 13)

As the first congener of the acyclic nucleoside analogs (ANPs), (*S*)-HPMPA could be envisaged as resulting from hybridization of DHPA with phosphonoacetic acid; it afforded a broad-spectrum activity against various DNA viruses [38]. For its sister compound, PMEA, activity was noted against retroviruses [38,39]. After Yokota et al. had demonstrated its activity against hepatitis B virus (HBV) [40,41], adefovir (PMEA) was further evaluated by Marcellin et al. [42], and was launched as adefovir dipivoxil (Hepsera^®^) by Gilead Sciences for the treatment of chronic HBV infections.

**Figure 11:**
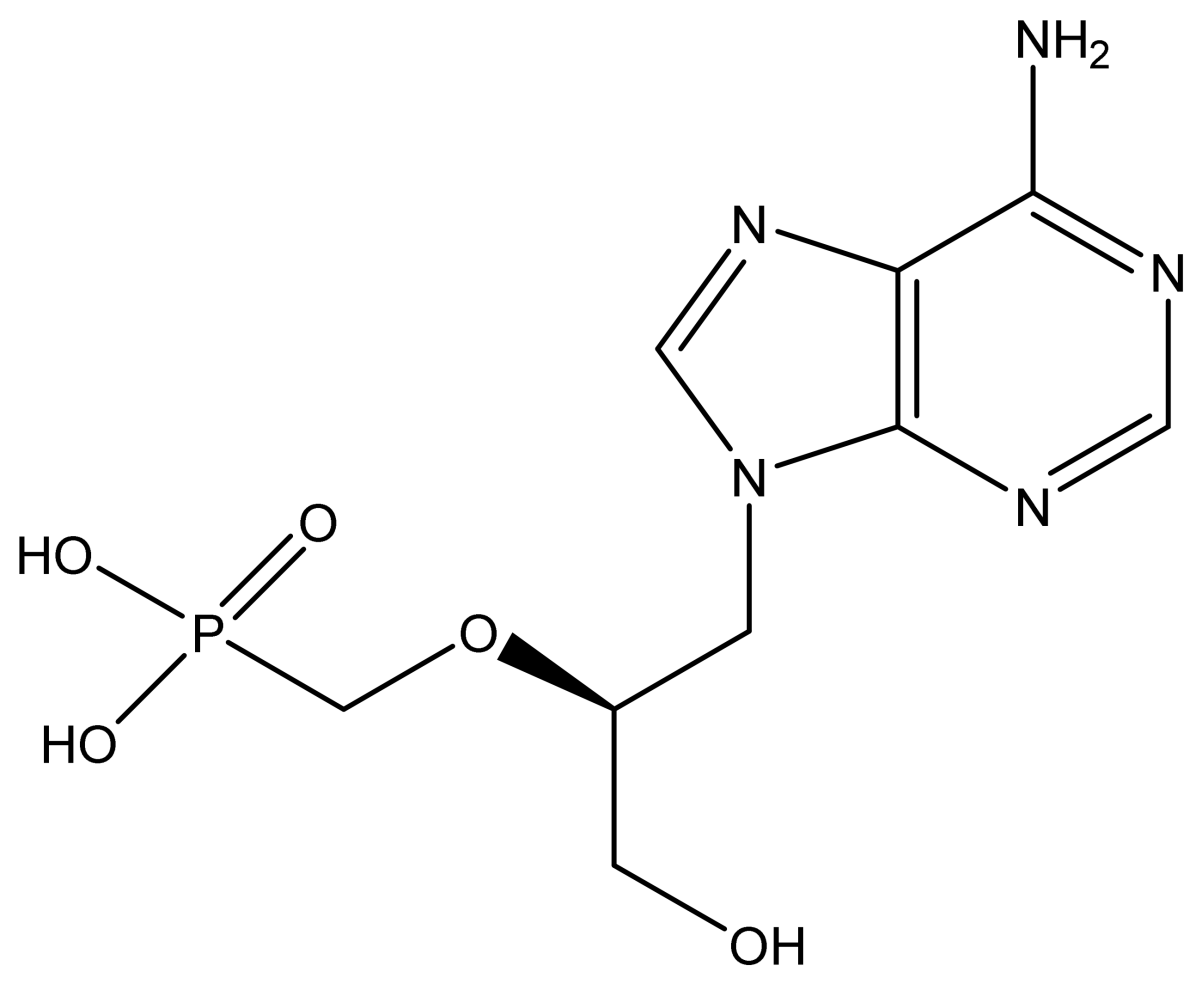
HPMPA.

**Figure 12:**
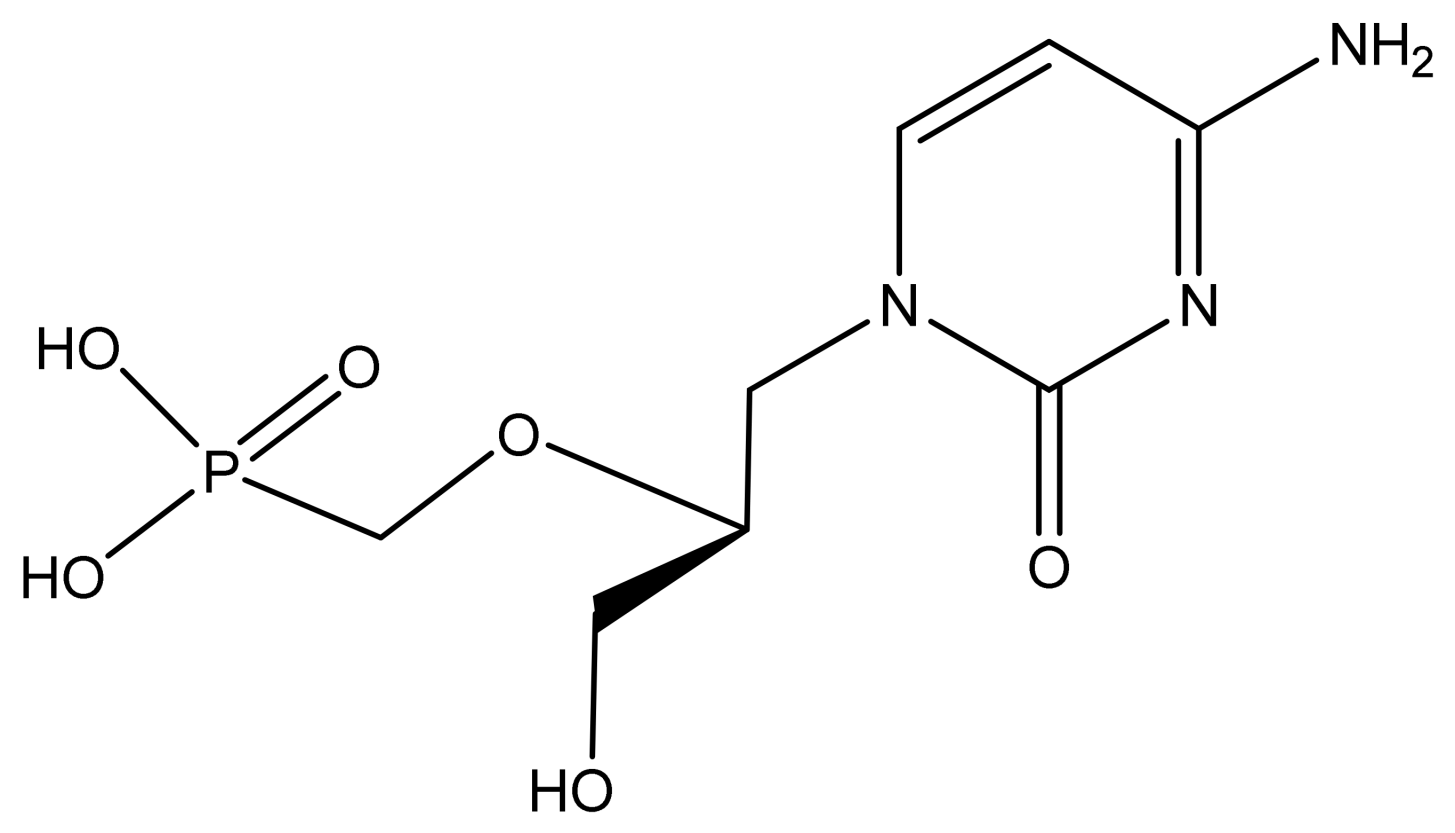
HPMPC, Cidofovir, Vistide^®^.

**Figure 13:**
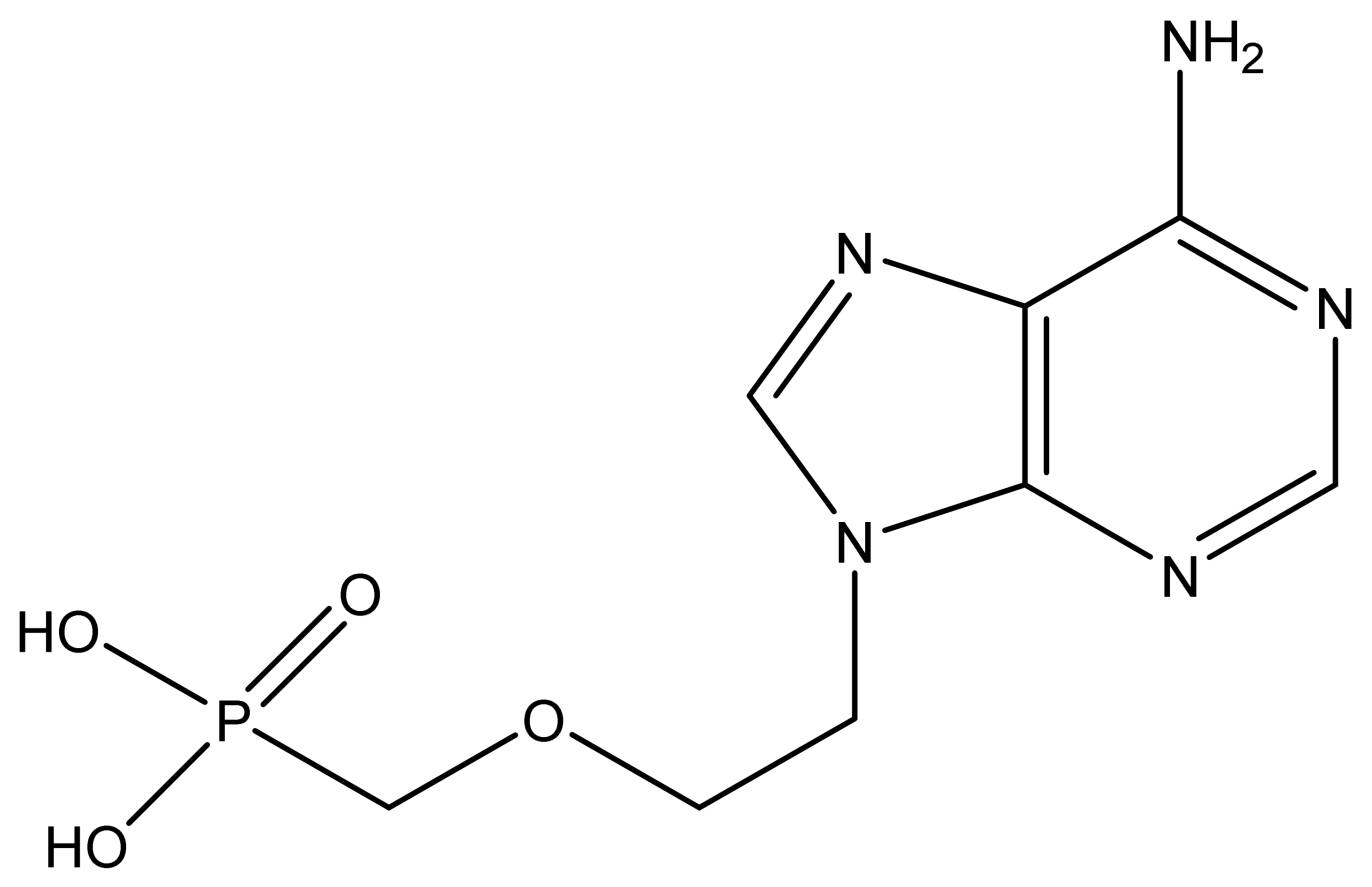
PMEA, Adefovir, Adefovir dipovoxil, Hepsera^®^.

Although anecdotal case reports pointed to the clinical efficacy of (*S*)-HPMPA in the treatment of adenovirus infections (i.e., keratoconjunctivitis), it was never commercialized for this purpose. Gilead Sci. preferred to develop (*S*)-HPMPC (cidofovir) over (*S*)-HPMPA after Snoeck et al. [43] had demonstrated its activity against human cytomegalovirus (CMV) in cell culture. After the necessary safety studies had been completed and Lalezari et al. [44] had demonstrated its clinical efficacy, cidofovir (Vistide^®^) was eventually marketed in 1996 for intravenous administration in the treatment of CMV retinitis in AIDS patients.

Although it was not formally approved for any of these indications, cidofovir (Vistide^®^) has been used successfully on a compassionate basis for the treatment of various DNA virus infections, i.e., pharyngeal papillomatosis [45], laryngeal papillomatosis [46], pulmonary papillomatosis [47], and cervical intracellular neoplasia (CIN) [48], that may subsequently evolve to carcinoma of the cervix uteri, molluscum contagiosum [49] and orf (ectyma contagiosum or sheep pox) [50]. Cidofovir has proven effective in the treatment of various human papilloma virus (HPV) infections [51,52]. Other case studies point to the efficacy of cidofovir in the topical treatment of common HPV infections such as warts.

## 14. Tenofovir [(R)-PMPA] (Figure 14)

At Gilead Sciences they first aimed at acquiring FDA approval for launching PMEA (adefovir) for the treatment of HIV infections. There were at least three issues that argued against this initial planning: (*i*) PMEA was rather toxic at the envisaged dosage for treating HIV infections, (*ii*) a new ANP analog [(*R*)-PMPA] that was not toxic at higher doses had in the meantime been developed, and (*iii*) adefovir in its oral prodrug form, adefovir dipivoxil (Hepsera^®^), would be suitable for use in the treatment of HBV infections at a much lower dosage than originally envisaged for HIV infections.

**Figure 14:**
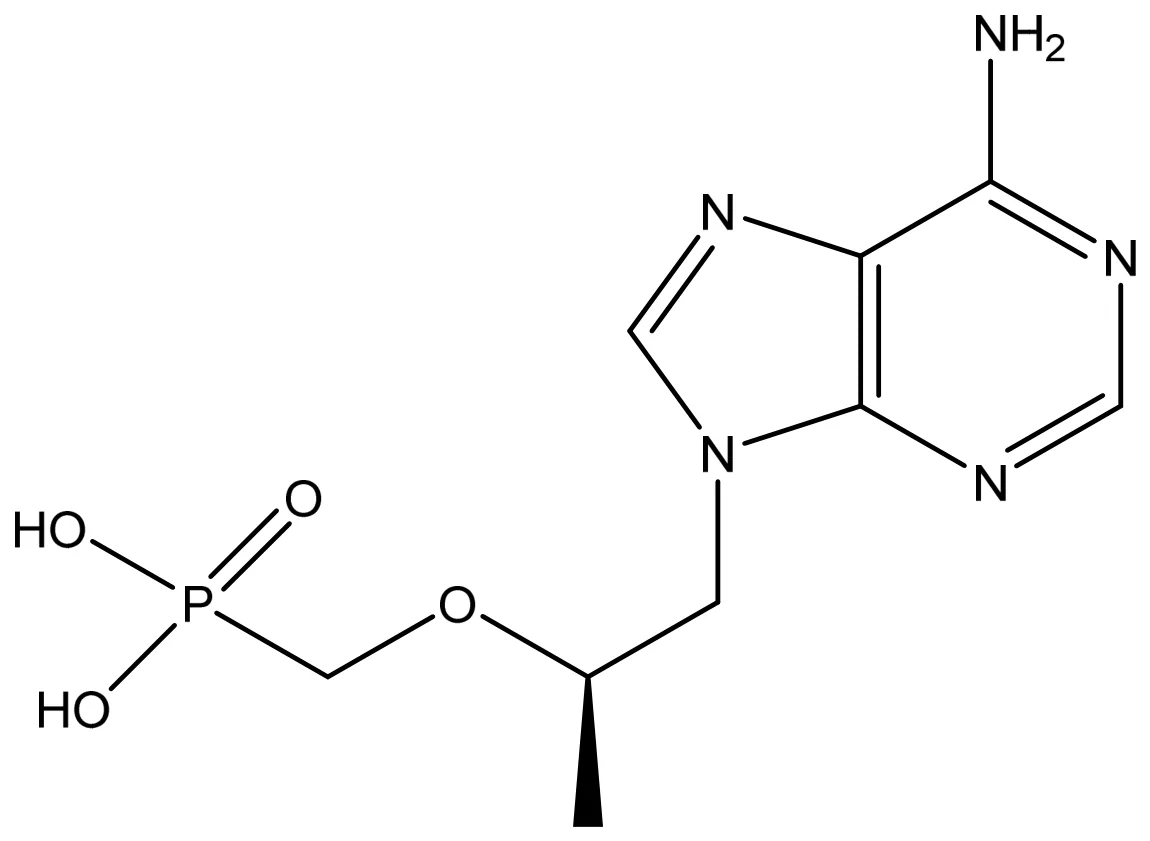
PMPA, tenofovir disoproxil fumarate (TDF), Viread^®^.

[(*R*)-PMPA] had originally been described in 1993 by Balzarini et al. [53] for its anti-HIV activity, and Tsai et al. [54] had demonstrated in 1995 that it completely blocked simian immunodeficiency virus (SIV) infection in rhesus macaques if administered shortly before or after the virus challenge. In retrospect, this observation presaged that tenofovir might be able to prevent HIV infections, and, hence, it could be viewed as the prelude to what later should be termed pre-exposure prophylaxis (PrEP) of HIV infections. Akin to adefovir, tenofovir was converted to its disoproxil derivative to ascertain its oral bioavailability [55,56], and the latter was then formulated as its fumarate, and launched by Gilead in 2001 as Viread^®^ (TDF, tenofovir disoproxil fumarate) for the treatment of HIV infections.

**Figure 15:**
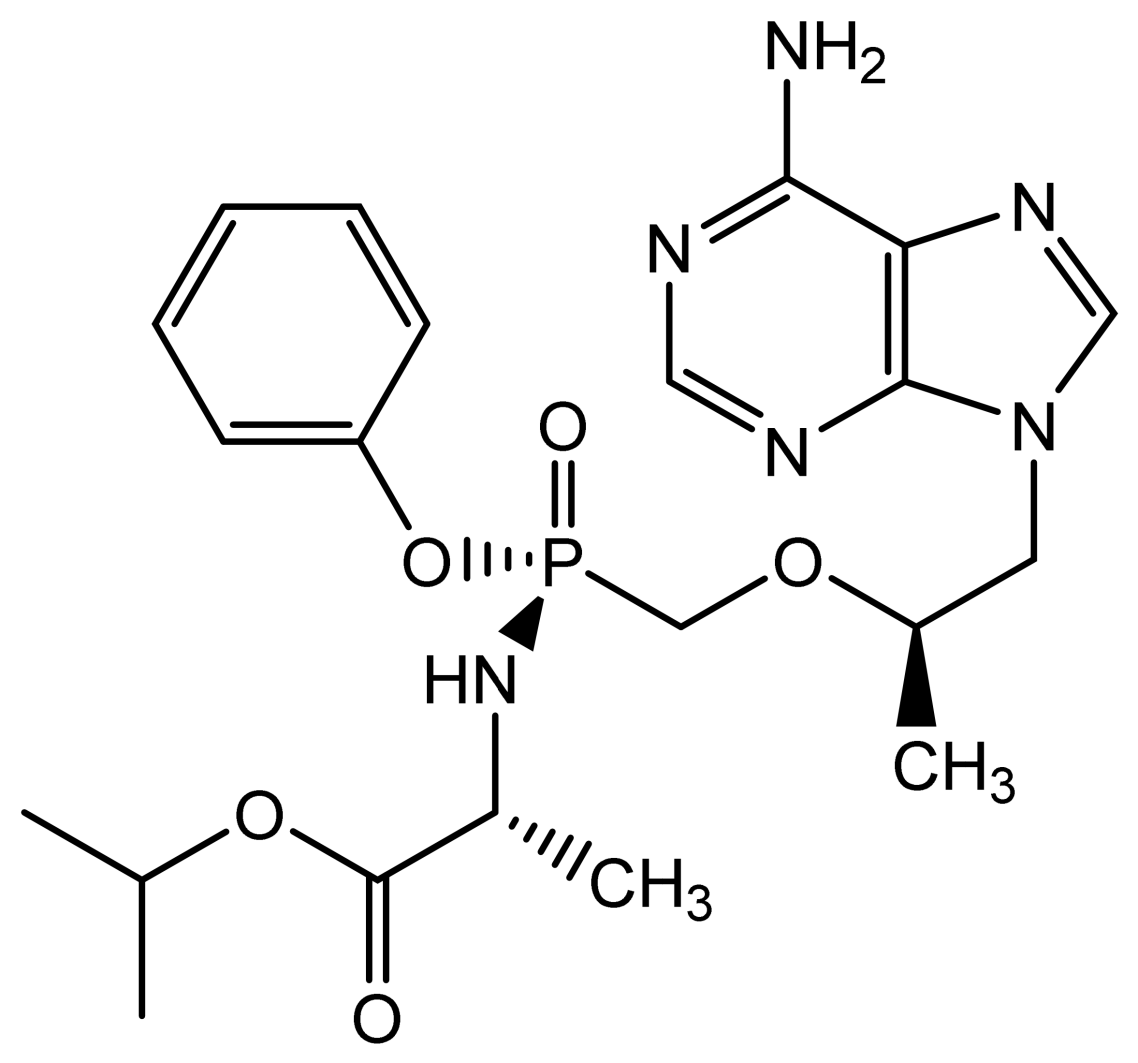
Tenofovir alafenamide (TAF).

In combination with emtricitabine [Emtriva^®^, (−)FTC] TDF was approved (launched) as Truvada^®^ for the treatment of HIV infections. This combination was extended to Atripla^®^ (combination of TDF, (−)FTC and efavirenz) in 2006, Complera^®^/Eviplera^®^ (combination of TDF, (−)FTC and rilpivirine) in 2011, and Stribild^®^ (combination of TDF, (−)FTC, elvitegravir and cobicistat) in 2012. Meanwhile, a new tenofovir derivative (TAF, tenofovir alafenamide) was developed by Lee et al. [57] (Figure 15), which, later on, was combined with several other compounds for the treatment of HIV infections [58]. Akin to the combination of TDF with (−)FTC (Truvada^®^), the combination of TAF with (−)FTC (Descovy^®^) has been approved for the prevention of HIV infections (PrEP), and TAF (Vemlidy^®^) has also been launched for the treatment of HBV infections.

## 15. 1-[(2-Hydroxyethoxy)methyl]-6-(phenylthio)thymine (HEPT) (Figure 16) and Tetrahydroimidazobenzodiazepinone (TIBO) Derivatives (Figure 17)

The HEPT derivatives were originally synthesized by Hiromichi Tanaka in the lab of Tadashi Miyasaka (Showa University, Japan), and forwarded to me through the aid of R.T. Walker as possible anti-HSV agents. As pyrimidine analogs they had, as I expected, no anti-HSV activity, but totally unexpected was that one of the HEPT derivatives showed anti-HIV activity. When Dr. Masanori Baba found this activity in our lab, we did not have the slightest hint as to its mode of action, and nevertheless we published our (preliminary) findings in *Biochemical and Biophysical Research Communications* [59] and the *Journal of Medicinal Chemistry* [60].

**Figure 16:**
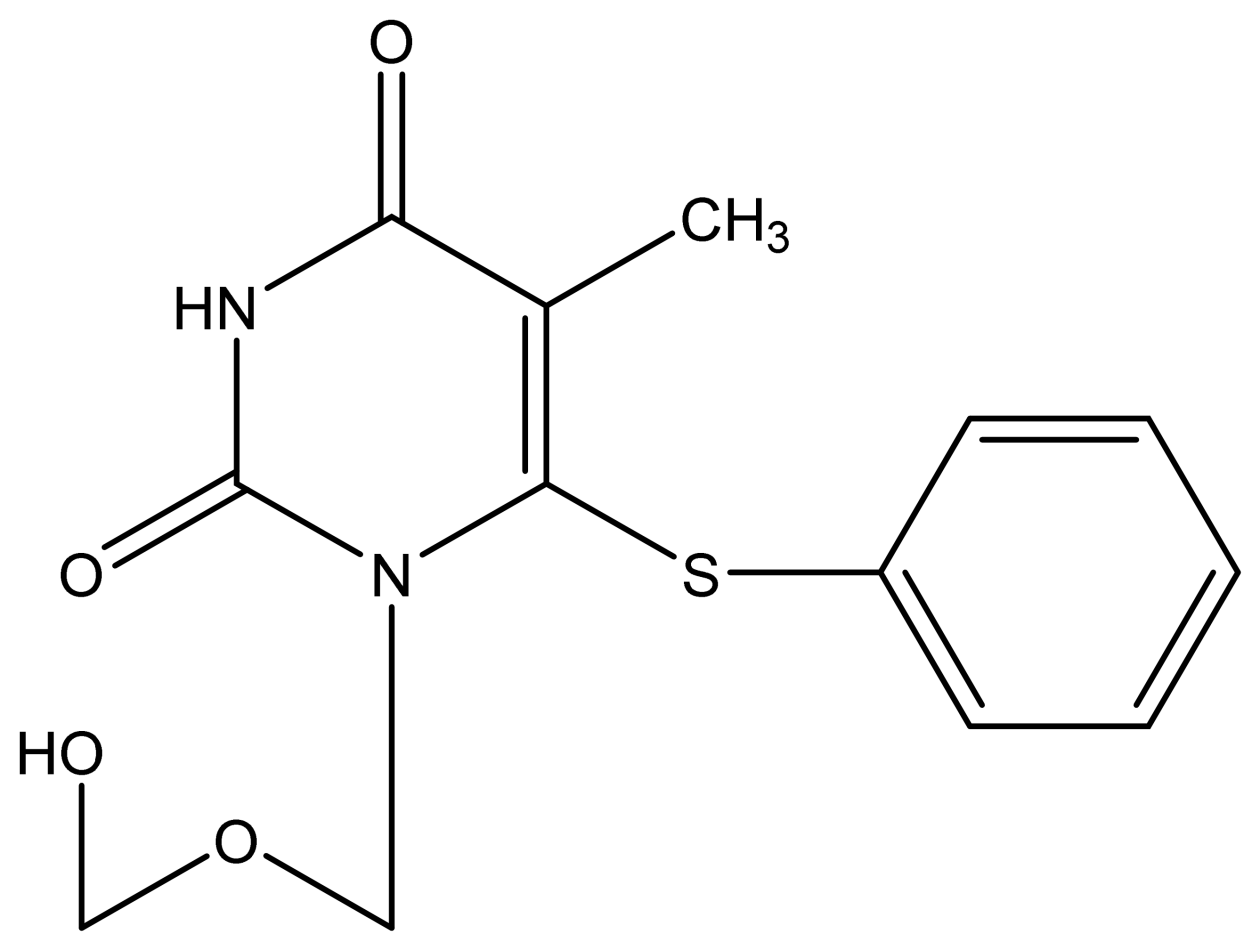
HEPT.

**Figure 17:**
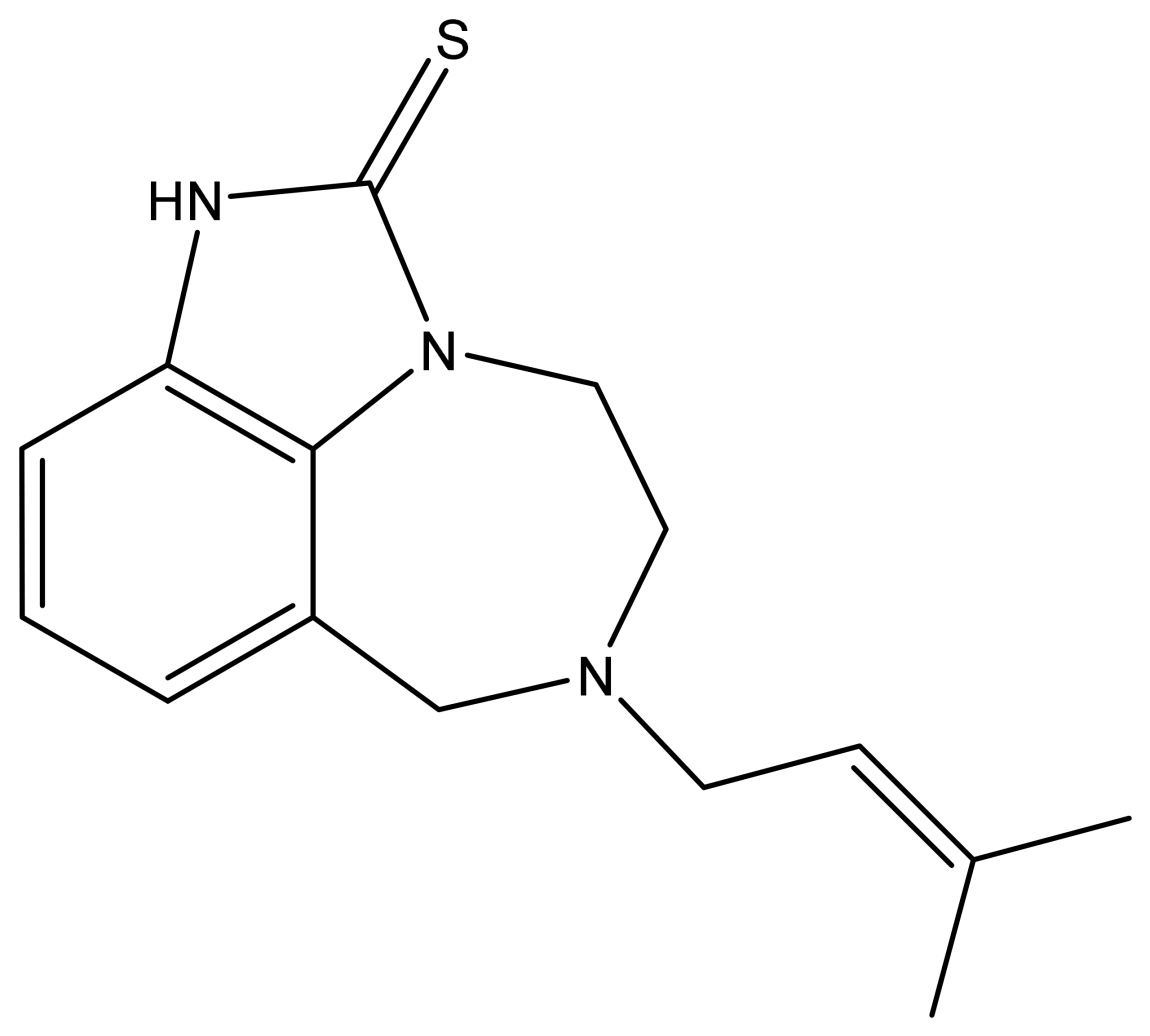
TIBO.

Simultaneously with the HEPT derivatives we had evaluated the TIBO derivatives, and in this case, thanks to the “time of addition” experiment elaborated by Rudi Pauwels, we had suggestive evidence that the TIBO derivatives achieved specific activity against HIV-1 due to an interaction with the reverse transcriptase [61]. That the TIBO derivatives were targeted at the HIV-1 RT was further ascertained by Debyser et al. [62].

For the HEPT derivatives it became increasingly clearer that they followed a similar strategy as the TIBO derivatives, i.e., an interaction with the HIV-1 RT [63,64]. Although it was hard to visualize a similarity in the structural conformation of the HEPT and TIBO derivatives, I constructed a possible concept that would reconcile the two divergent origins in the discovery of specific anti-HIV-1 RT activity [65].

From the HEPT derivatives, emivirine [66] was identified as the clinical candidate (Coactinon^®^) that moved from Burroughs Wellcome to Triangle Pharmaceuticals and finally, Gilead Sciences where its further development was discontinued.

The TIBO derivatives followed a meandrous path leading to rilpivirine (RPV) [67] that was finally formulated in conjunction with cabotegravir (CAB), to be administered by intramuscular injection once per 2 months (Q2M) to prevent HIV infection [68].

## 16. HIV Protease Inhibitors (PIs)

In total, ten PIs were marketed as anti-HIV agents: saquinavir (Figure 18), ritonavir (Figure 19), indinavir (Figure 20), nelfinavir (Figure 21), amprenavir (Figure 22), lopinavir (Figure 23), atazanavir (Figure 24), fosamprenavir (Figure 25), tipranavir (Figure 26) and darunavir (Figure 27).

**Figure 26:**
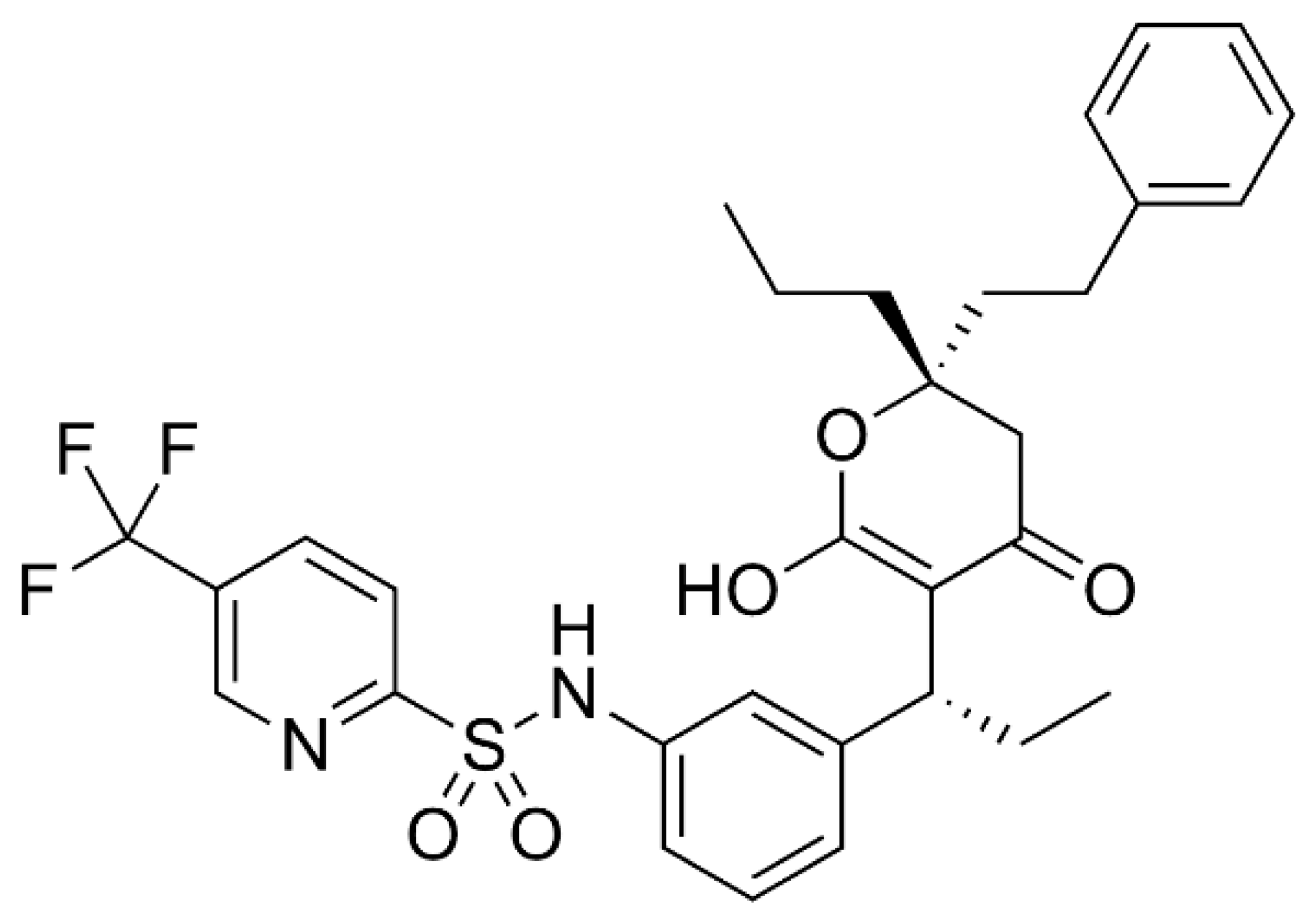
Tipranavir, Aptivus^®^.

With the exception of tipranavir (Figure 26), all PIs can be considered as “peptidomimetic”, containing a hydroxyethyl group instead of the peptide linkage. Consequently, they inhibit breakage of the peptide linkage(s) which should be cleaved during the maturation of the HIV viral capsid protein(s).

## 17. HIV Integrase Strand Transfer Inhibitors (INSTIs)

**Figure 28:**
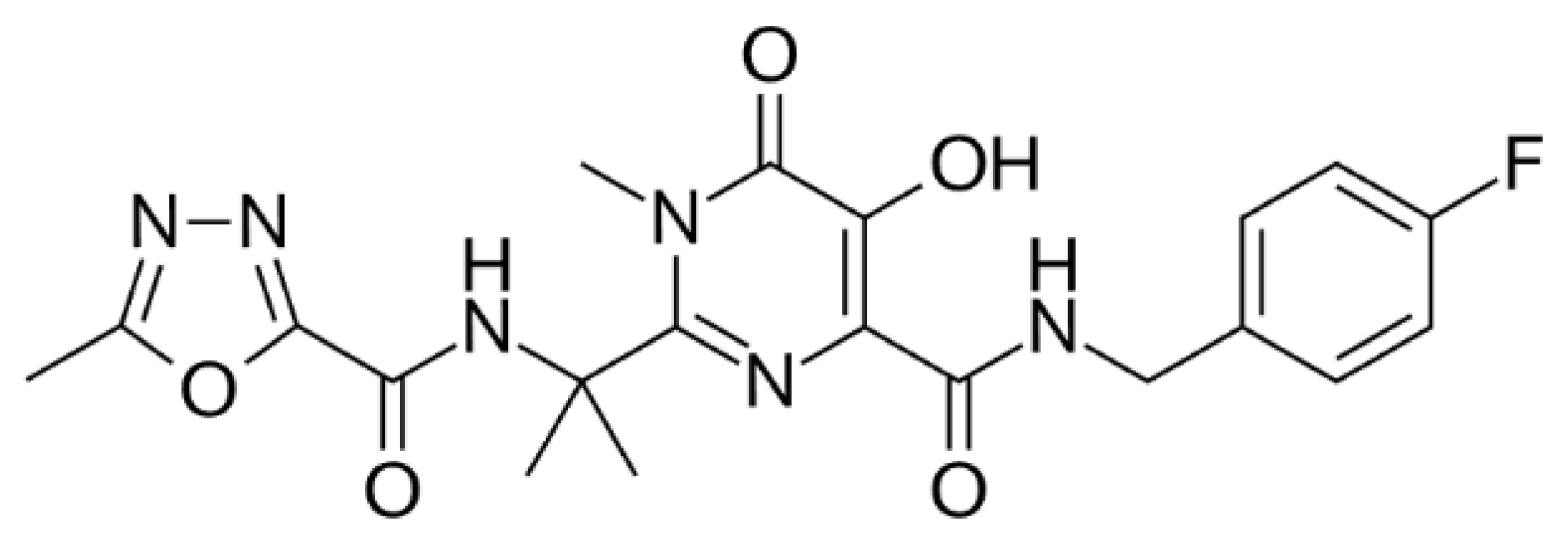
Raltegravir, Isentress^®^.

**Figure 29:**
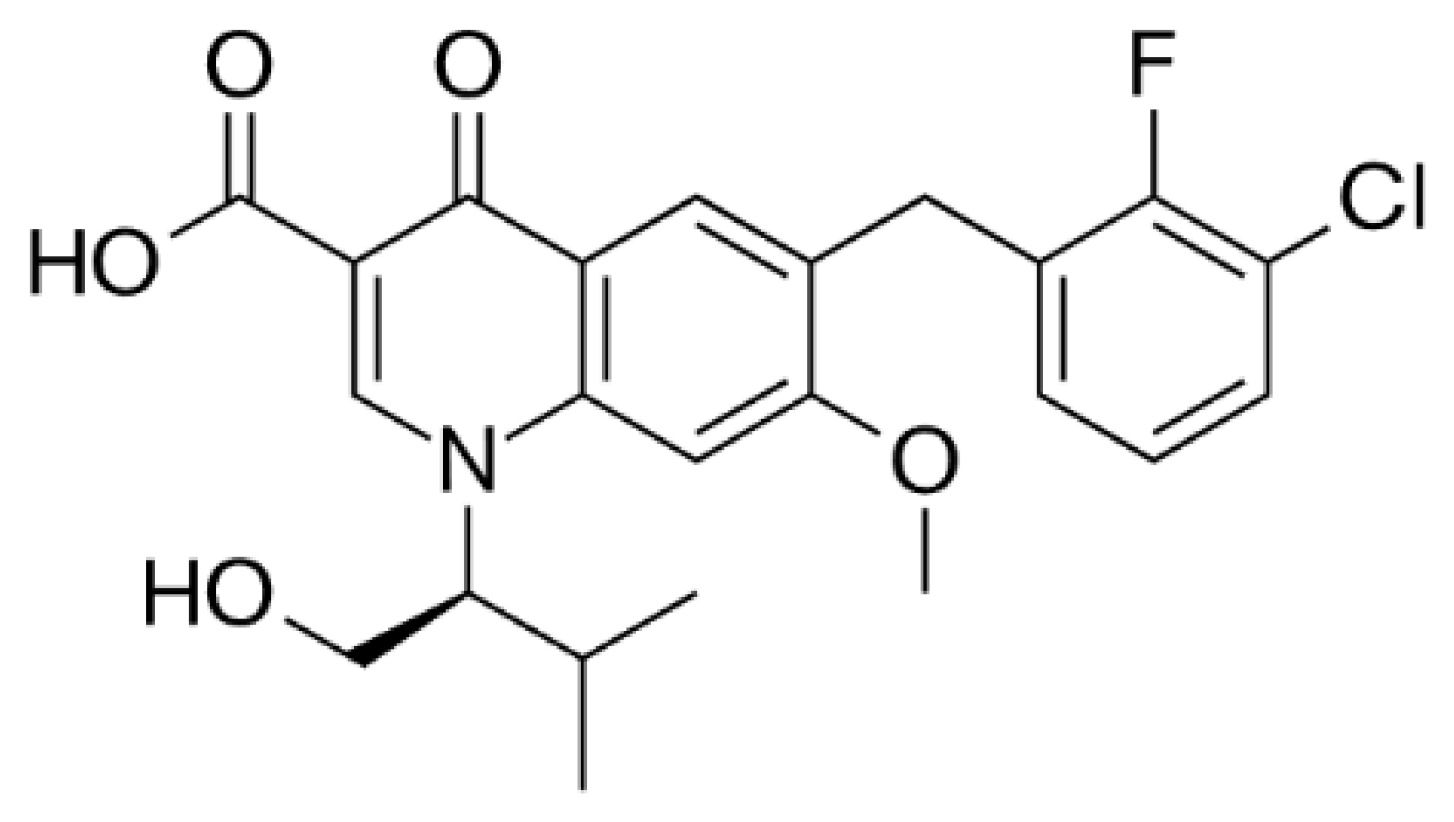
Elvitegravir, Stribild^®^.

**Figure 30:**
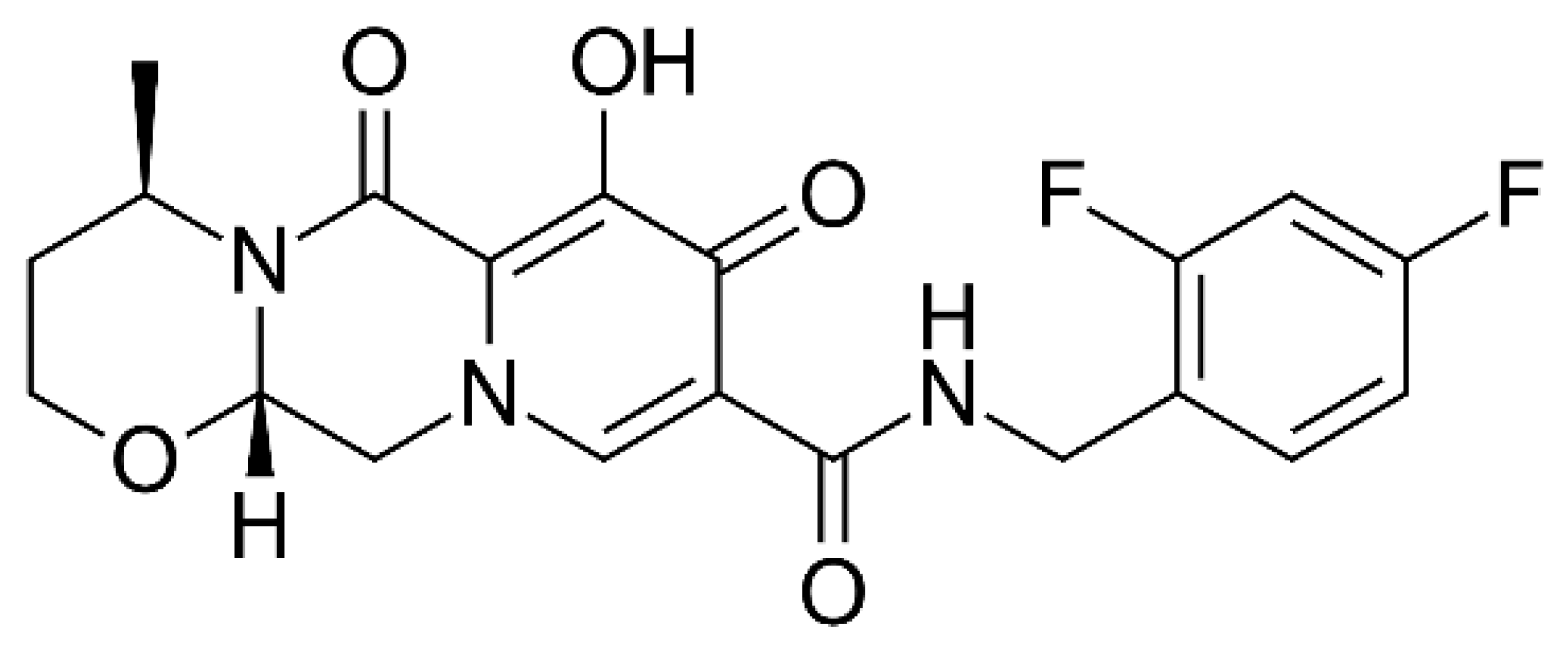
Dolutegravir, Tivicay^®^.

**Figure 31:**
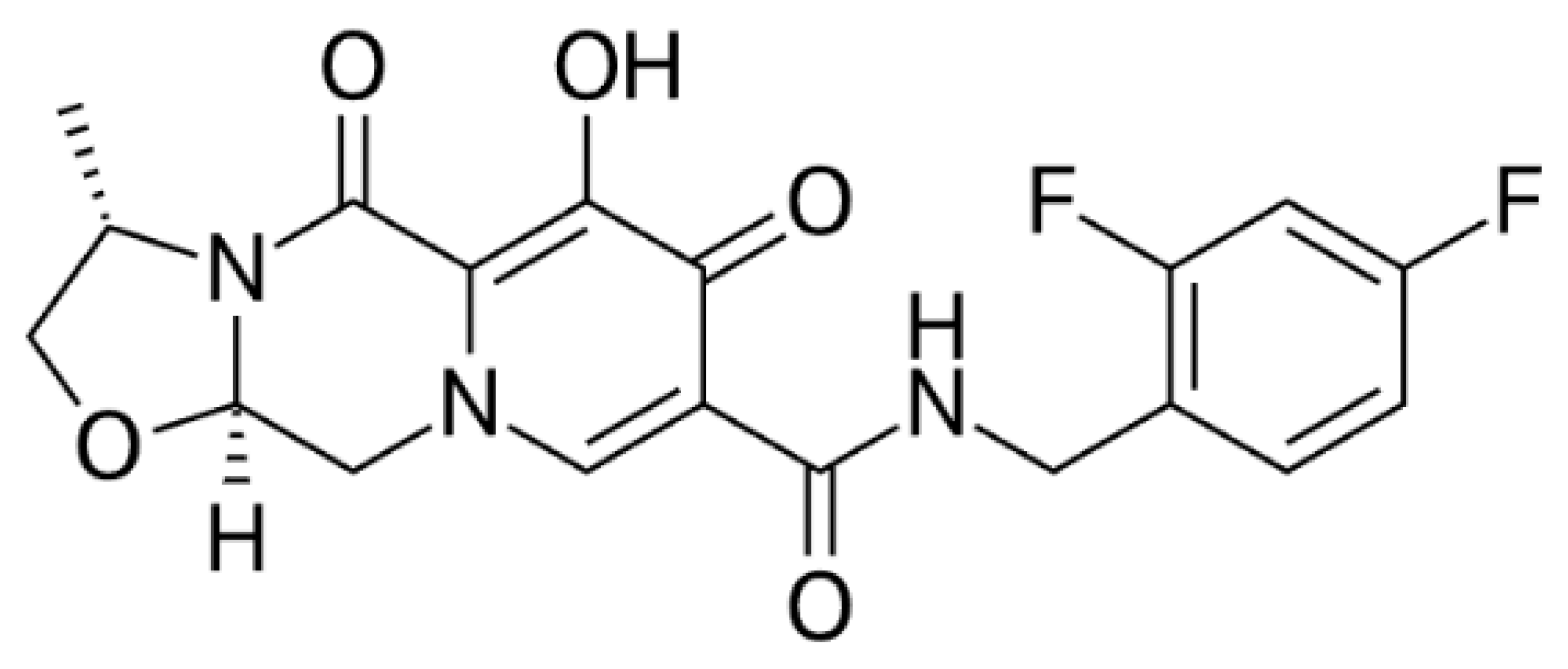
Cabotegravir, Vocabria^®^, Apretude^®^.

After the HIV RNA has been converted by the viral RT into proviral DNA, the latter can be integrated into the cellular DNA. Four INSTIs have been recognized to inhibit this process: raltegravir (Figure 28), elvitegravir (Figure 29), dolutegravir (Figure 30) and cabotegravir (Figure 31). As mentioned above [68], cabotegravir can be administered parenterally Q2M together with rilpivirine to prevent HIV infection.

## 18. HIV Capsid Inhibitor Lenacapavir

With one injection every six months or even once a year it would now seem feasible to prevent HIV infection [69]. This opens new perspectives for the eventual elimination of the disease (AIDS) as well as the underlying virus infection. A longer account of the potential of Lenacapavir is forthcoming.

## 19. The Stem Cell Mobilizer AMD-3100 (Figure 32)

The bicyclams were originally discovered as anti-HIV agents putatively targeted at the viral uncoating process [70]. A major increase in anti-HIV activity was observed with the bicyclam AMD-3100 [71]. In the initial preclinical studies, Dr. Craig Hendrix detected that AMD-3100 boosted the white blood cell (WBC) counts, which in subsequent studies were primarily identified as CD34^+^ or hematopoietic stem cells. Thus, the anti-HIV agent appeared to act as a stem cell mobilizer, and the successive steps characterizing this process, have been repeatedly reviewed [72,73,74,75,76].

**Figure 32:**
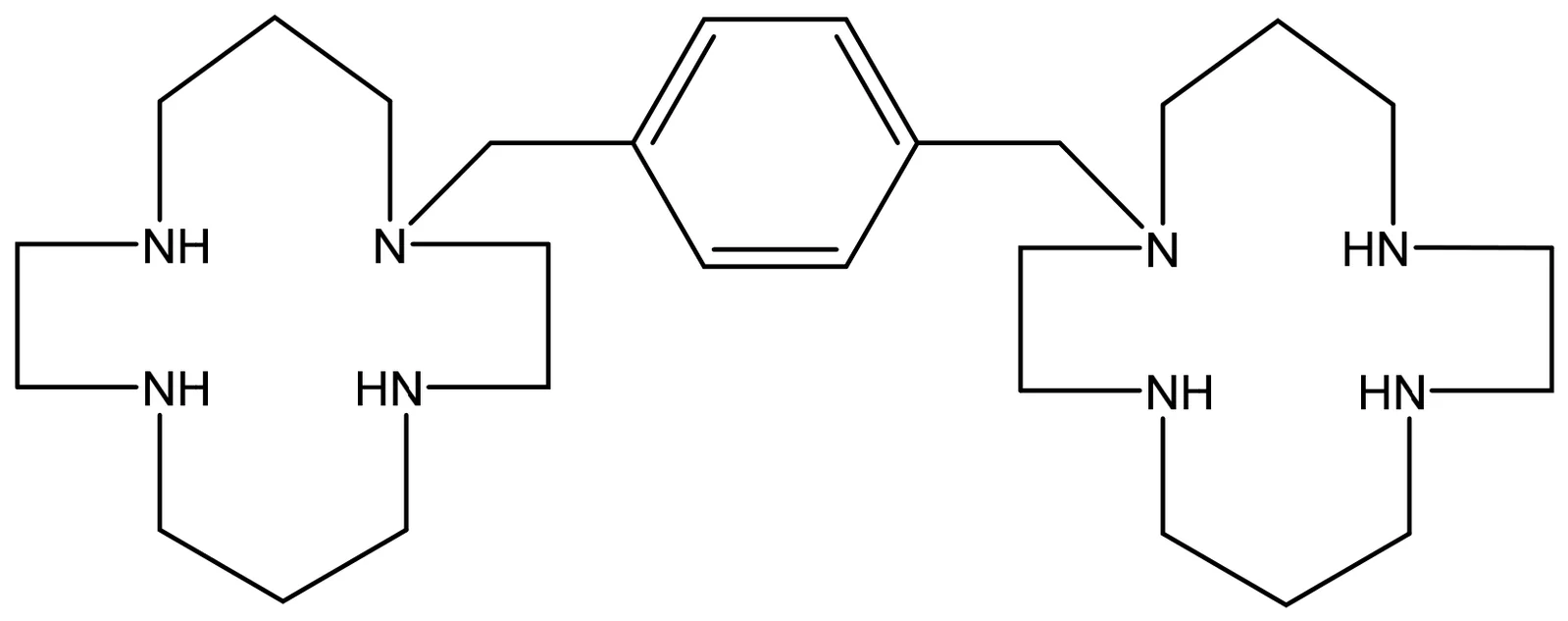
AMD-3100, Plerixafor, Mozobil^®^.

As a stem cell mobilizer (AMD-3100, Mozobil^®^) has found its major therapeutic application in autologous stem cell transplantation in patients with non-Hodgkin lymphoma (NHL) or multiple myeloma (MM). Other indications remain to be identified. Plausible utility is certainly expected in the treatment of hereditary immunological disorders such as WHIM syndrome, and physiopathological processes such as hepatopulmonary syndrome (HPS) [76].

## 20. Annus Horribilis: 2006

In 2006, I had the misfortune to become 65 years old on 28 March 2006. As if I was not already aware of this mishap, I was reminded by the rector of the KU Leuven that at the age of 65, I had to be stripped of all my functions, including (*i*) services in committees such as the “beoordelingscommissie” to judge the suitability of new candidate professors and the “evaluatiecommissie” (for which I was even chosen as chairman) to evaluate whether the appointed professors fulfilled the assumed duties. That I was relieved of these functions did not come as a surprise because of the imposed terms foreseen. More difficult to accept (or digest) was the termination of my research work (*ii*), enforced by the shut-off of all financial support and technical assistance. Even more difficult, and totally unacceptable was the fact that I had to stop teaching (*iii*), a duty in which I got more experienced by aging, allowing me to distinguish more important from less important facts to teach the students. This duty was irrevocably finished at the KU Leuven in 2006, but still allowed for 3 years at the affiliated campus in Kortrijk (KULAK), where it was terminated in 2009, after a 37-year-long career of uninterrupted teaching at this campus.

In anticipation of what was going to happen in 2006, Dr. Antonín Holý had already advised me in 2005 that I should come to Prague to enjoy a more comfortable “fin de carrière”. I finally did so in teaching my beloved course on “Biochemistry at the service of Medicine”, under the guidance of Prof. Libor Grubhoffer, for interested bachelor/master students at Jihoceska University in Česke Budéjovice from 2007 till 2023. In the last two years of my teaching, it shifted from “ex cathedra” to electronically, and this electronic teaching was further implemented in Changsha in China in 2025 and 2026 for my teaching of a course on “Viruses and Antivirals”.

Since 2006, I have been entitled to carry the title of “emeritus”, although I doubt whether this title should be regarded as a promotion (as it was originally intended), or allowance (as it commonly signifies), or disqualification (which it basically means in reality).

## 21. A New Discovery: Guangdi Li (“Lowie”) in 2014

In August 2014, Guangdi Li (“Lowie”) successfully defended his PhD thesis with Prof. Annemieke Vandamme as adviser. I was an accidental participant at the ensuing reception, from which an unedited student–professor relationship emanated between Lowie and myself, that gave rise to an abundance of joint publications: first, (*i*) publications devoted to antiviral drugs in general [77] and their basic principles [78], (*ii*) strategies to conquer hepatitis C virus (HCV) infections [79], and (*iii*) concepts for curtailing coronavirus (SARS-CoV-2) infections [80,81]. How to deal with dengue viruses and hemorrhagic virus infections is the subject of forthcoming publications.

## 22. Perspectives

While approaching the 3000th publication on specific antiviral agents, a number of prospects still herald future perspectives. (*i*) Although highly potent and selective in its anti-VZV activity, BVDU could still be surpassed in potency and selectivity by the valine ester of Cf1743 (FV-100) [82]. (*ii*) As a stem cell mobilizer, the utility of AMD-3100 (plerixafor, Mozobil^®^) could be envisaged for various diseases [other than non-Hodgkin lymphoma (NHL) and multiple myeloma (MM)] where stem cells contribute to a successful healing process [76]. (*iii*) Lenacapavir [69] and the Q2M combination of cabotegravir (CAB) with rilpivirine (RPV) [68], while efficacious in the prevention of HIV infections, enable the (re-)emergence of HBV infections; this offers the opportunity for TAF and (−)FTC which have been recognized as anti-HBV agents. (*iv*) The growing incidence of hemorrhagic fever virus infections, such as Ebola and others [83,84], should necessitate the development of specific antiviral compounds coping with these viruses. (*v*) Cidofovir (Vistide^®^) has been approved solely for the treatment of CMV retinitis in immunosuppressed (i.e., HIV-infected) patients. It also deserves further pursuit for the treatment of HPV infections (i.e., pharyngeal, laryngeal, or cervical carcinoma) because of the beneficial results obtained in initial anecdotal studies.

## 23. Epilogue

Although interferon owes its name to “interference” with viruses and their propagation, it was in the mid-70s temporarily envisaged as a potential antitumor agent. That role was then taken over by the reverse transcriptase (RT). It led me to believe that suramin, which I had just identified as an RT inhibitor, could exhibit an inhibitory effect on cancer, i.e., leukemia, in mice. This hope appeared to be unjustified. Instead, suramin showed anti-HIV activity and thus opened the search for equally (or more) potent (and less toxic) anti-HIV agents, leading to the NRTIs, NNRTIs, PIs and INSTIs.

That AIDS could eventually be eradicated is no longer an unrealistic dream, but that the causative virus in its proviral DNA form may be apt to elimination, is akin to DNA viruses, herpes simplex virus or other herpesviruses, an apparently unfeasible task.

Besides those I have already mentioned, I wish to thank the late Antonín Holý and his colleagues at IOCB in Prague, the late John C. Martin and his colleagues at Gilead Sciences, Prof. Dr. Libor Grubhoffer (Ceske Budejovice) as the scientific successor of A. Holý, Prof. Dr. Guangdi Li (“Lowie”, Changsha, China) as my own scientific successor, and my son Prof. Dr. Rafael De Clercq (Lingnan University, Hong Kong) for their continuous advice and encouragement.

## Figures and Tables

**Figure 18:**
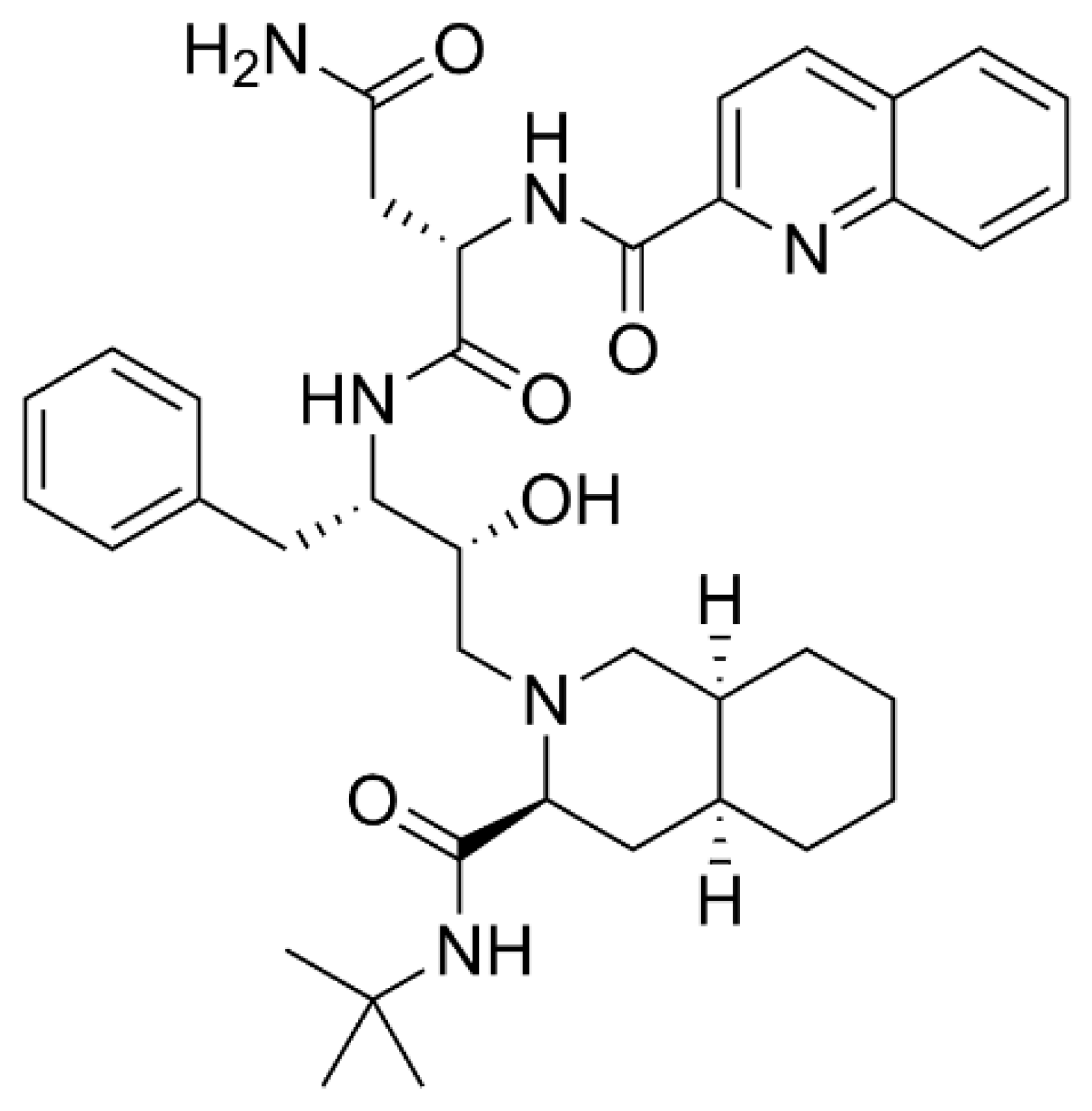
Saquinavir, Invirase^®^, Fortovase^®^.

**Figure 19:**
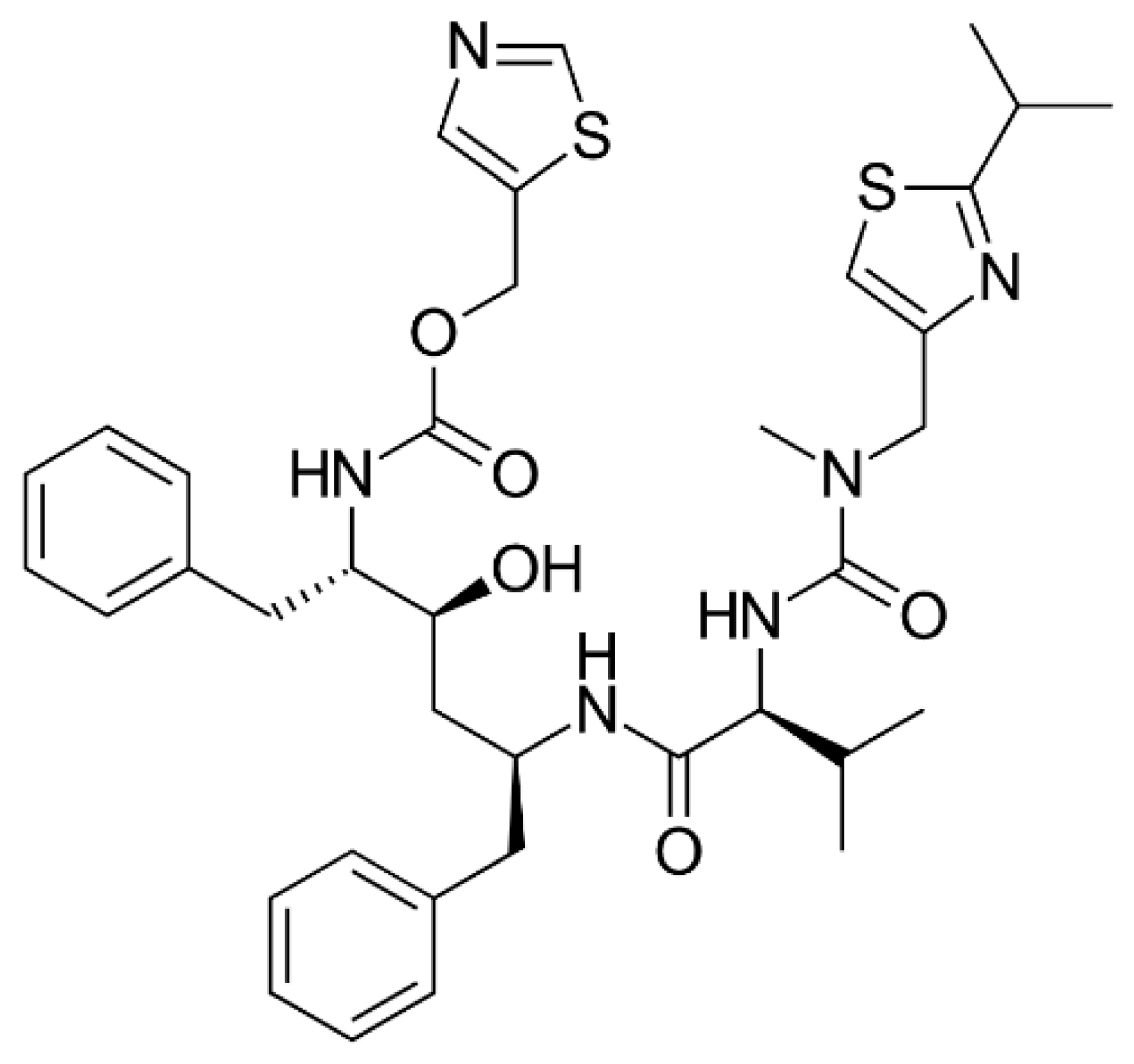
Ritonavir, Norvir^®^.

**Figure 20:**
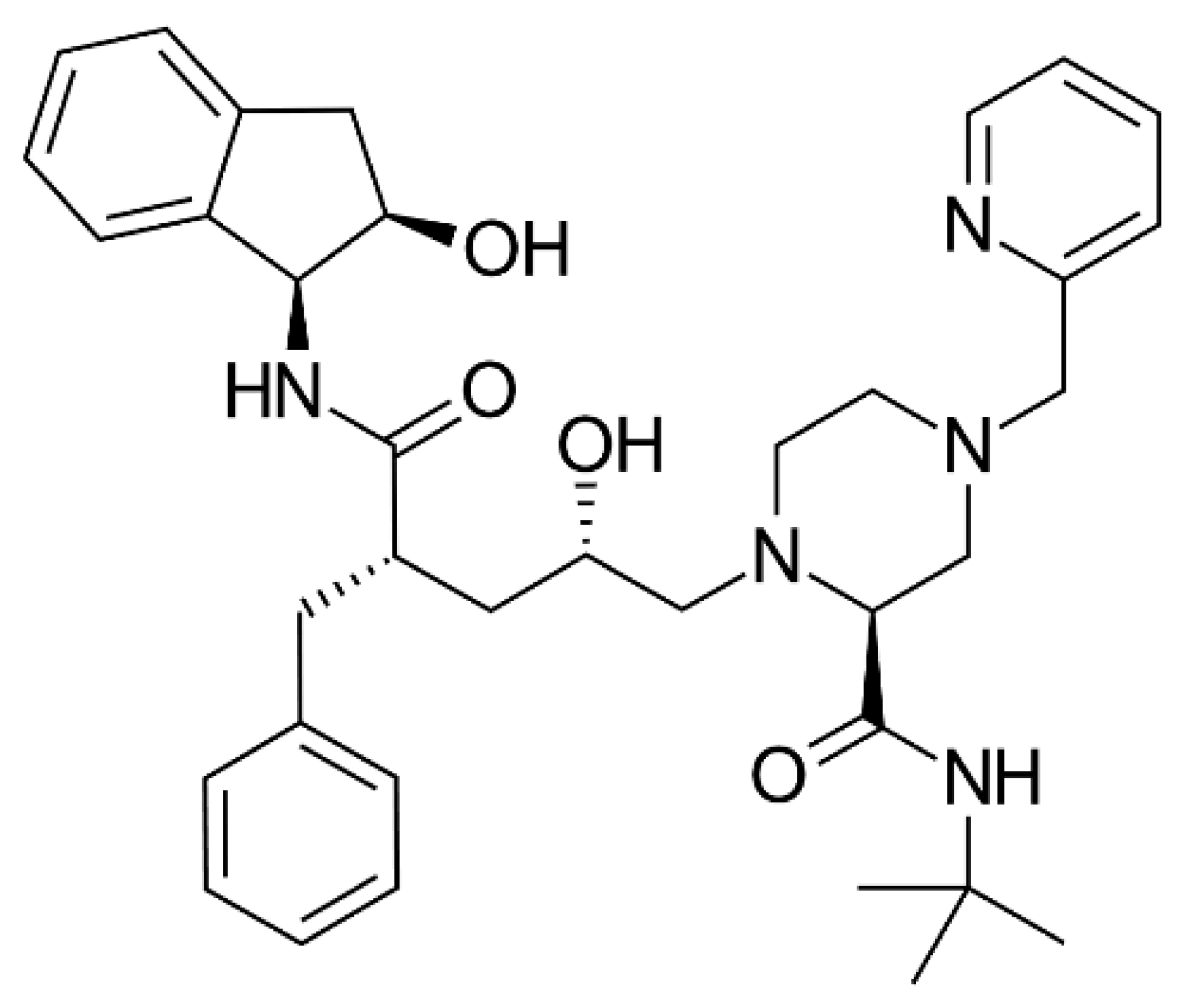
Indinavir, Crixivan^®^.

**Figure 21:**
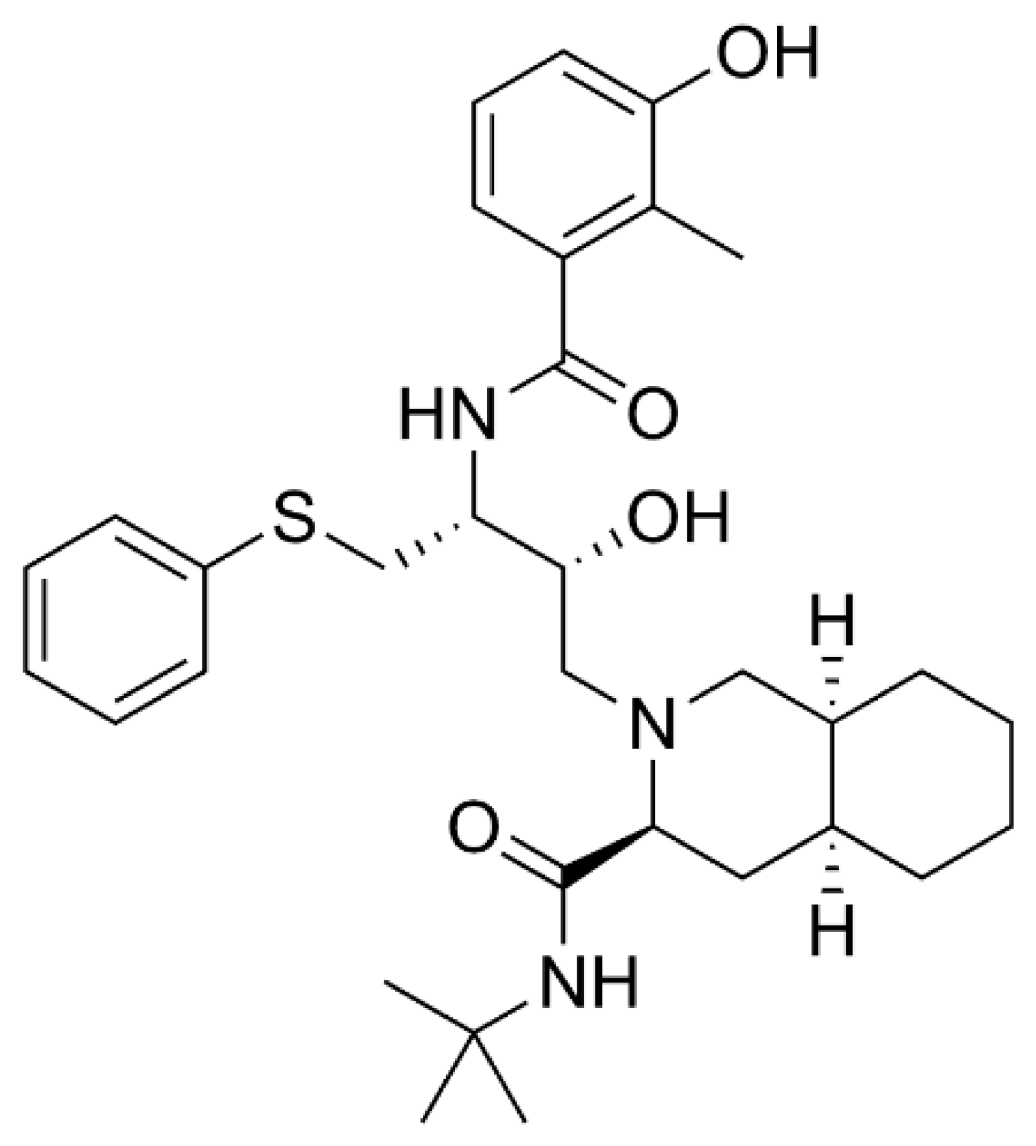
Nelfinavir, Viracept^®^.

**Figure 22:**
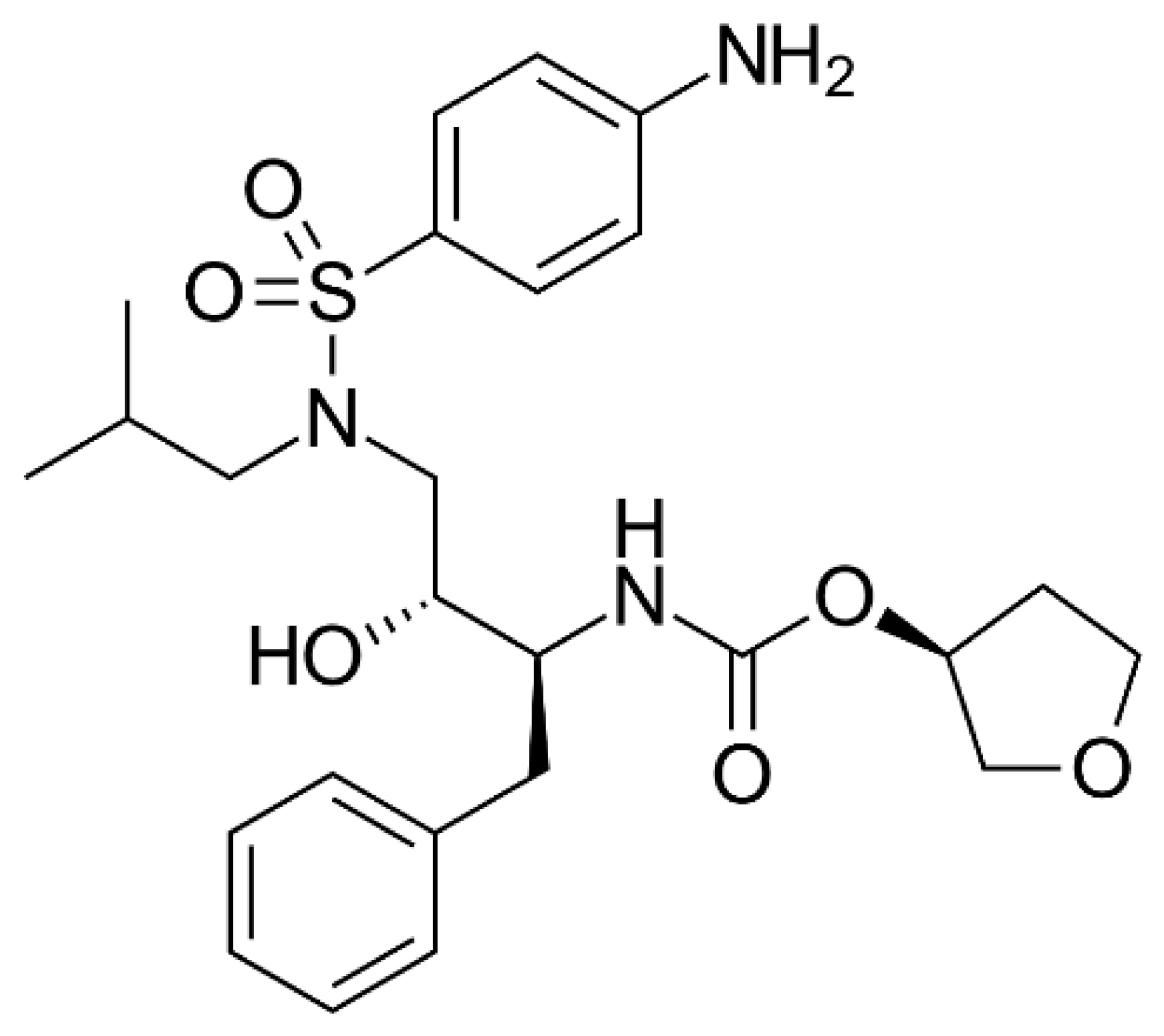
Amprenavir, Agenerase^®^.

**Figure 23:**
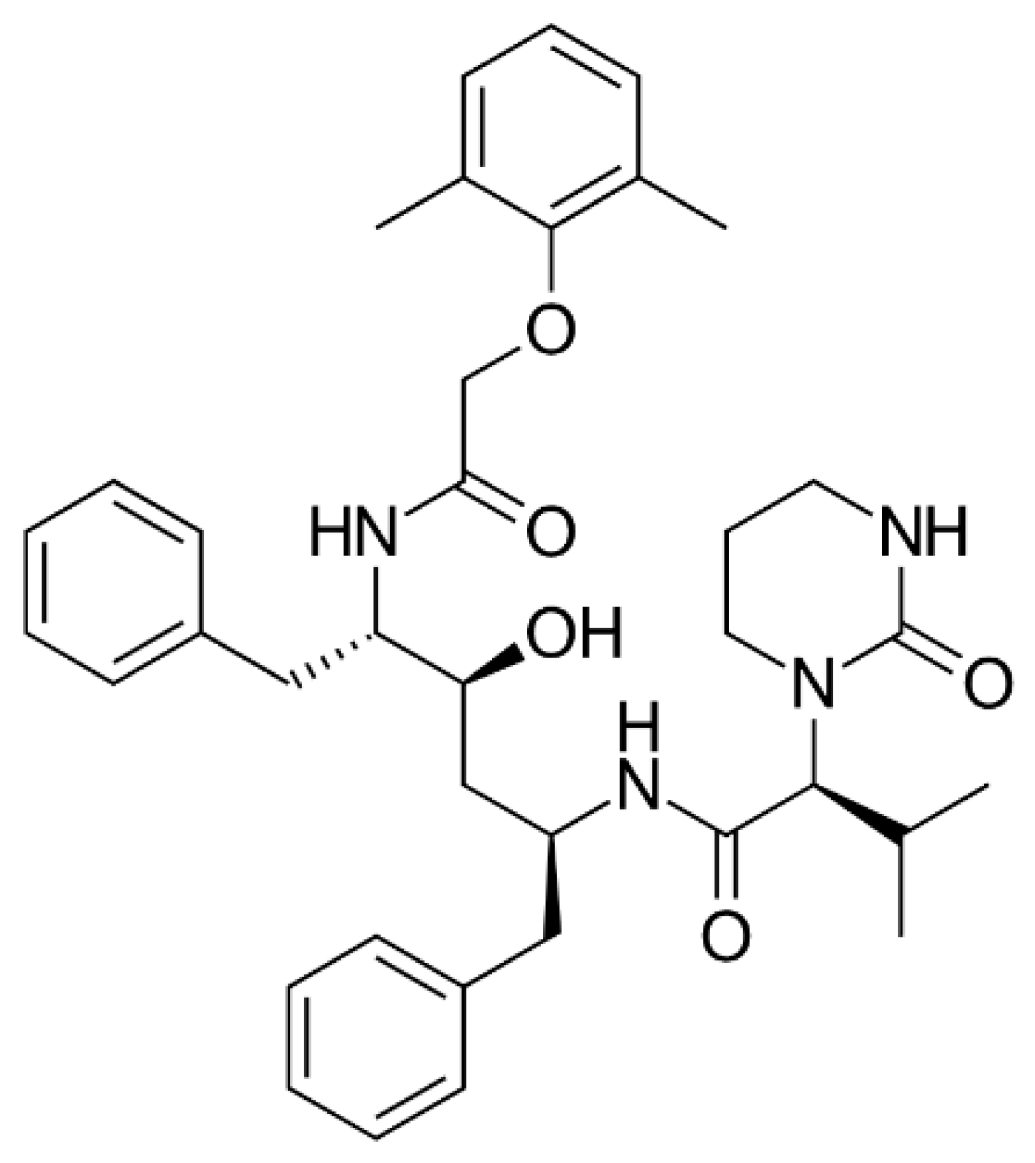
Lopinavir.

**Figure 24:**
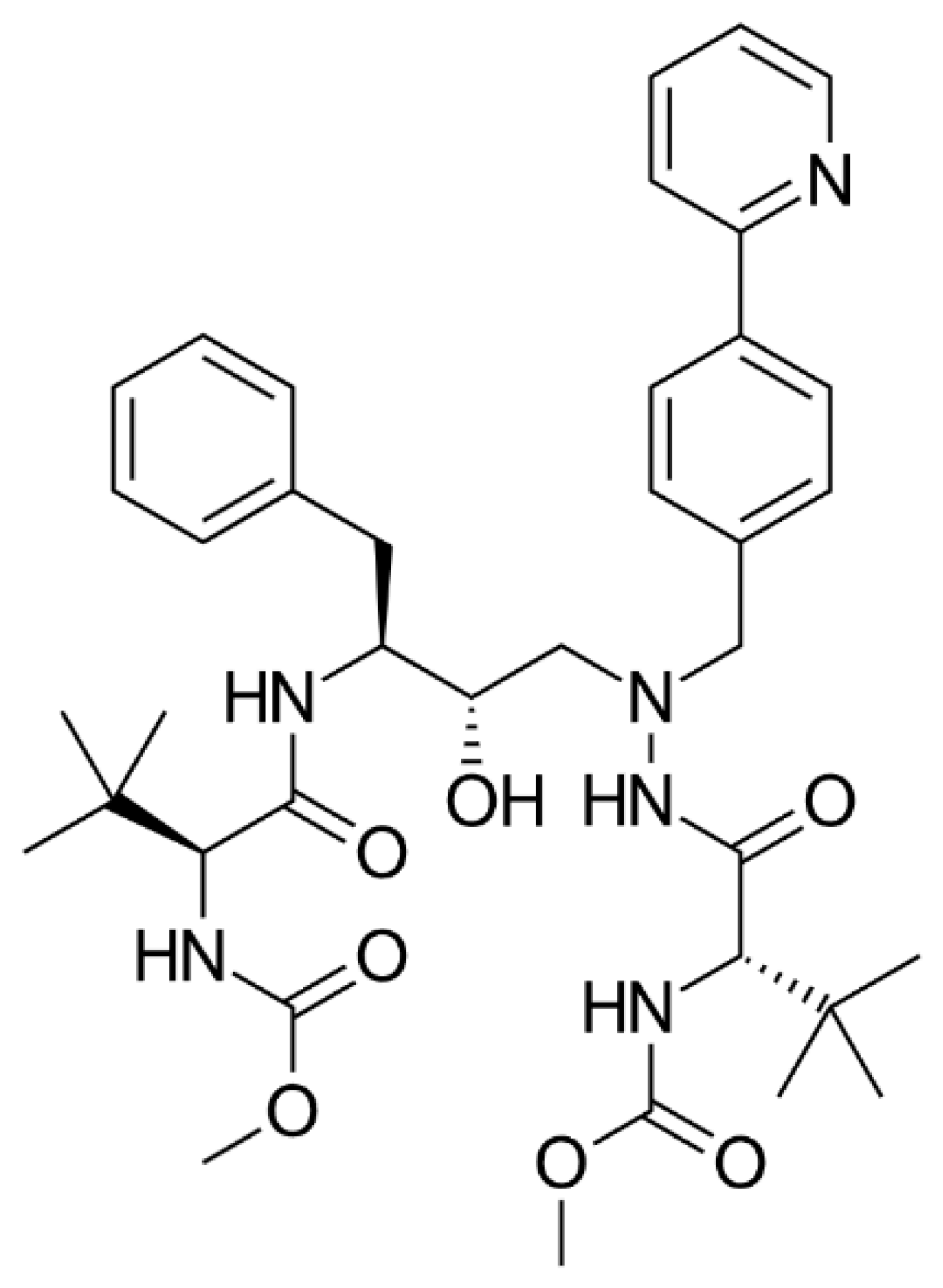
Atazanavir, Reyataz^®^, Evotaz^®^, ….

**Figure 25:**
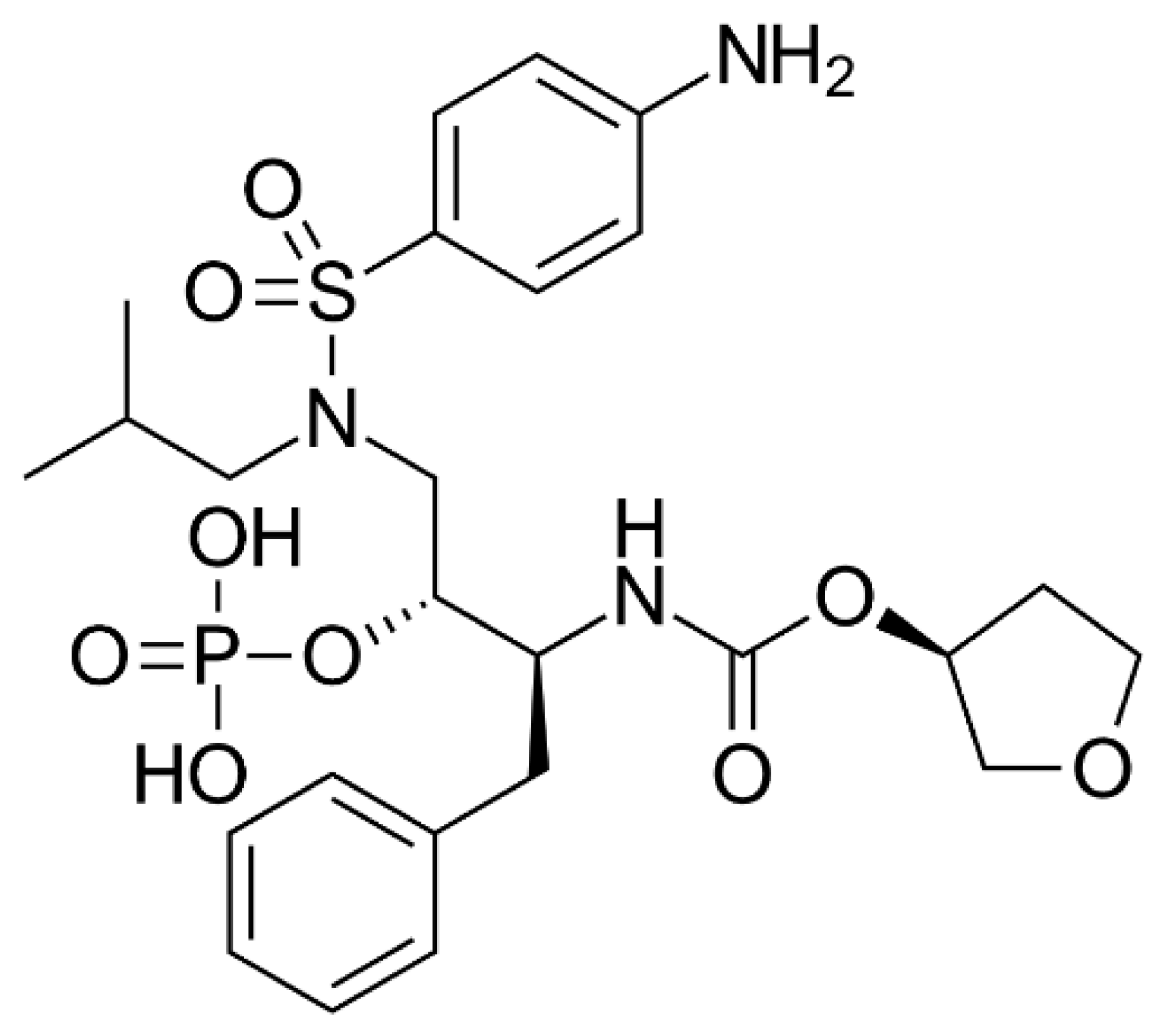
Fosamprenavir, Lexiva^®^, Telzir^®^.

**Figure 27:**
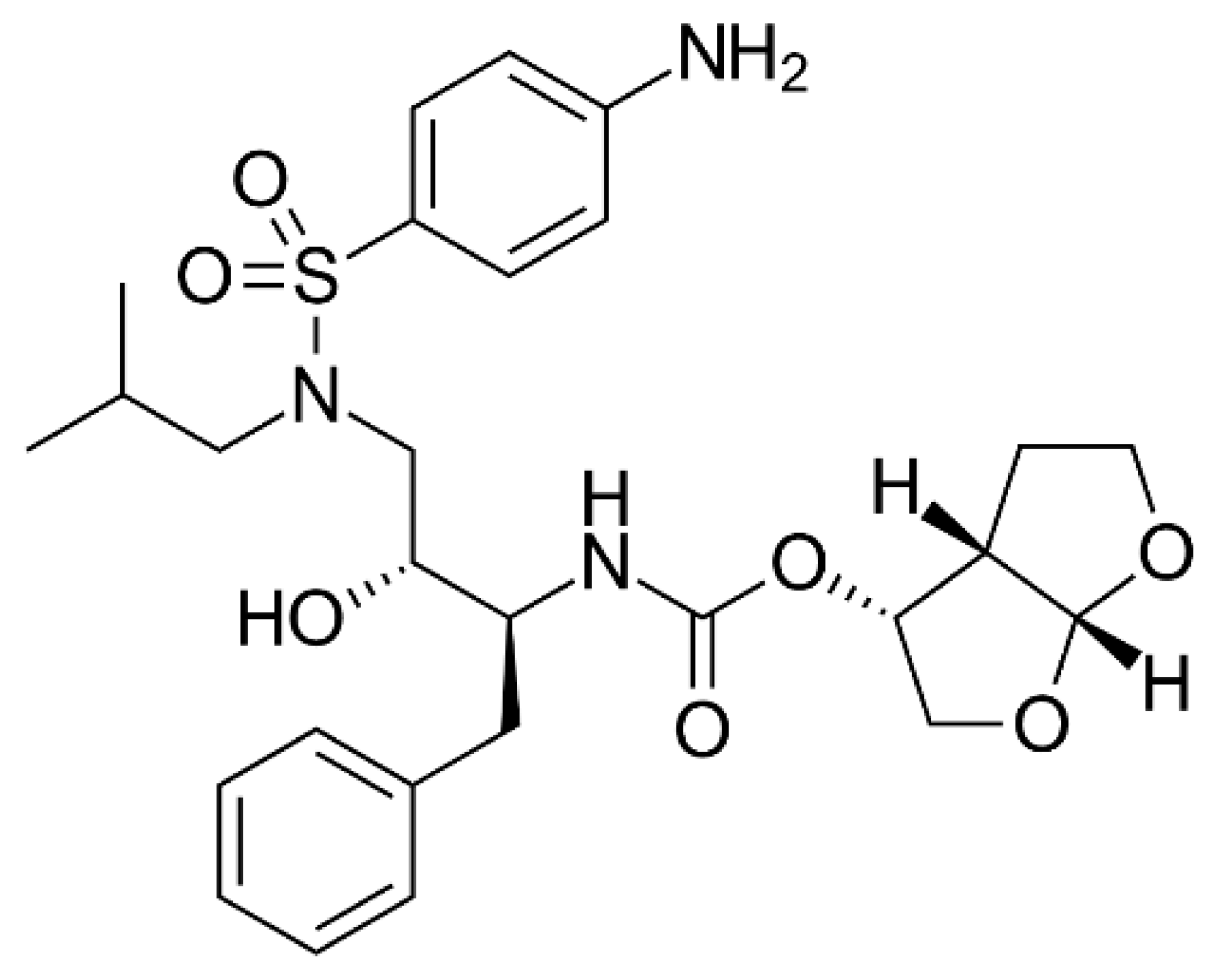
Darunavir, Prezista^®^, ….

## Data Availability

No new data were created or analyzed in this study. Data sharing is not applicable to this article.
